# Reptiles of Chubut province, Argentina: richness, diversity, conservation status and geographic distribution maps

**DOI:** 10.3897/zookeys.498.7476

**Published:** 2015-04-21

**Authors:** Ignacio Minoli, Mariana Morando, Luciano Javier Avila

**Affiliations:** 1 Grupo de Herpetología Patagónica, CENPAT-CONICET, Boul. Almt. G. Brown 2915, U9120ACD, Puerto Madryn, Chubut, Argentina

**Keywords:** Biogeography, diversity, herpetofauna, conservation, central Patagonia, Argentina

## Abstract

An accurate estimation of species and population geographic ranges is essential for species-focused studies and conservation and management plans. Knowledge of the geographic distributions of reptiles from Patagonian Argentina is in general limited and dispersed over manuscripts from a wide variety of topics. We completed an extensive review of reptile species of central Patagonia (Argentina) based on information from a wide variety of sources. We compiled and checked geographic distribution records from published literature and museum records, including extensive new data from the LJAMM-CNP (CENPAT-CONICET) herpetological collection. Our results show that there are 52 taxa recorded for this region and the highest species richness was seen in the families Liolaemidae and Dipsadidae with 31 and 10 species, respectively. The Patagónica was the phytogeographic province most diverse in species and *Phymaturus* was the genus of conservation concern most strongly associated with it. We present a detailed species list with geographical information, richness species, diversity analyses with comparisons across phytogeographical provinces, conservation status, taxonomic comments and distribution maps for all of these taxa.

## Introduction

Precise estimation of species’ geographic ranges based on accurate taxonomic identification is central for species-focused studies and conservation and management plans (Feeley and Silman 2011, Katzner et al. 2011). Knowledge of reptile diversity in southern areas of Argentina has increased considerably in recent decades through numerous published monographs and books (Gallardo 1971, Cei 1973b, 1986, Scolaro 2005, 2006, Avila et al. 2006b, Abdala 2007). However, information on reptile geographic distributions, as well as systematic and ecological aspects is still limited, especially for large areas with difficult access, which remain unsurveyed. The current distribution knowledge of reptiles of Chubut province is fragmented, with data deriving from original species descriptions, geographic citations in the form of short notes, partial reviews or phylogenetic and phylogeographic studies (Abdala 2002, 2003, 2005, Cei et al. 2003, Scolaro 2003, Scolaro and Ibargüengoytía 2007, Avila et al. 2008, Pincheira-Donoso et al. 2008, Lobo et al. 2010). Several studies have made contributions to the herpetological knowledge of this province Cei (1973a, 1975a, 1975b, 1978); Cruz et al. (1999); Daciuk and Miranda (1980); Etheridge and Christie (2003); Pincheira-Donoso and Núñez (2005); Scolaro (1976a, 1976b); Scolaro et al. (1985); Scolaro and Cei (1987) and Cei and Scolaro (1977, 1980, 1983, 1999, 2003), but only a few considered the conservation status, richness, diversity and accurate distribution of species (Breitman et al. 2014); which is essential information for conservation plans.

The northern and central areas of Patagonia have changed since the 1890s and have undergone steady change as a result of human activity, but there has been no clear understanding of the resulting effects on biodiversity. Over the twentieth century, business activities such as oil extraction, mining and ranching have caused changes in different ecosystems of this area. In particular, sheep overgrazing (Bisigato and Bertiller 1997, Cesa and Paruelo 2011) has led to a desertification process in the Monte and Patagonian Steppe ecoregions (Ares et al. 1995, Aguiar et al. 1996). Another factor that may affect the diversity and ecological dynamics of large xerophytic areas like this one (e.g., Schulze et al. 1996), is the creation of hydroelectric dams which implies anthropic management of regional water availability and seasonal changes in rainfall (Paruelo et al. 1998) or rivers flow rates (Masiokas et al. 2008). An overall analysis of reptile diversity and accurate species distributional data is essential information for understanding the impact and consequences of these types of human activity (Böhm et al. 2013, Cook et al. 2013).

Vertebrate surveys and the elaboration of regional lists provide basic information, not only for systematic and biogeographic studies, but also for wildlife conservation plans, natural management and bio-ecological studies. This study is the first reptile inventory with detailed and updated geographic distributional data for Central Patagonia, Chubut Province. We compiled and checked geographic distribution records from published literature and museum records, including extensive new data from the LJAMM-CNP (CENPAT-CONICET) herpetological collection. We performed a spatial analysis considering all sampled localities, and two species richness analyses: 1) related to sampled areas within a grid, and 2) related to phytogeographic provinces. Furthermore, we analyzed species diversity within phytogeographic provinces along with a dissimilarity index among them, and also detailed geographic information for reptile occurrence based on administrative (political) units called Departments. Additionally, we discuss all the geographic records considered erroneous or outdated on a separate taxonomic section.

## Materials and methods

### Study area

The study area of this work is comprised in the Chubut Province (Argentina), with a central-latitudinal location between 42°00'–46°00'S and 72°08'–63°35'W, covering approximately 224,686 km^2^ divided into 15 administrative departments (INDEC and IGM 2014). It has two clearly defined geographic regions: the Andean region confined to a narrow band on the west with granitic and metamorphic mountains; and an Extra-Andean region, characterized by volcanic terraces and plateaus product of volcanic events of the Tertiary and Quaternary (Scoppa 1998, Teruggi 1998). The climate is dry and cold in most of the territory, with an extremely variable temperature ranging, from -22.8 °C in winters to 41.3 °C degrees in summer (Teruggi 1998). The study area is characterized by four phytogeographic provinces: Patagónica, del Monte, Subantártica and Altoandina (Roig 1998). The majority of the field surveys were conducted in Patagónica and del Monte provinces and which have larger areas and higher numbers of presence records than the Subantártica province. The Altoandina province is the smallest in area and there are no reptile records from it, hence it was not represented on the map or included in the analyses.

### Methods

Extensive biological surveys began in early 1998 and continued until 2011, with field trips made at different representative areas of Chubut province. Most specimens were collected in the vicinity of roads and the majority of snake records are from individuals found killed by vehicles. Each record has a voucher number with a species identity assigned, date and place of origin. Collection sites were geographically referenced using a Garmin GPS 12™ Global Position Device. The systematic classification for families was according to Oppel (1811), Gray (1827, 1865), Frost et al. (2001), Gamble et al. (2008) and Zaher et al. (2009). The specimens were deposited in several herpetological collections: LJAMM-CNP (CONICET-CENPAT), BYU (Monte L. Bean Museum, Brigham Young University), MLP (La Plata Museum) and FML (Miguel Lillo Foundation). Additional museum collections and literature vouchered records were obtained from AMNH (American Museum of Natural History), CNHM (Chicago Natural History Museum; in the present The Field Museum of Natural History, FMNH), IADIZACH (Instituto Argentino de Investigaciones de las Zonas Áridas), JMCDC (Colección Herpetológica José Miguel Cei), CRILaR PT (Centro Regional de Investigaciones Científicas y Transferencia Tecnológica), MACN (Museo Argentino de Ciencias Naturales “Bernardino Rivadavia”), MCZ (Museum of Comparative Zoology, Harvard University), MHNG (Muséum d’histoire naturelle de la Ville de Genève), MZUC (Museo de Zoología de la Universidad de Concepción Chile), NMBA (Zoologische Expedition de Naturhistorischen Museums Basel), PT (Proyecto *Tupinambis*, Félix Benjamín Cruz), FBC (Félix Benjamín Cruz Field Collection), SDSU (San Diego State University), IBAUNC (Universidad Nacional de Cuyo), CH (Colección Centro Regional Universitario Bariloche, Universidad Nacional del Comahue, Río Negro, Argentina), MCN (Museo de Ciencias Naturales, Universidad Nacional de Salta) and UNMDP (Colección Herpetológica de la Universidad Nacional de Mar del Plata). Geographic information from the LJAMM-CNP collection and additional data from other collections and literature sources (see Institutions above, Supplementary file 1: Specimens examined) were considered for species presence analysis, according to Departmental units in Chubut province. Literature and museum records with acronyms or specific localities were quoted literally. We include these records from revisionary literature: Abdala (2005), Abdala (2007), Avila et al. (2001, 2003, 2006a, 2007a, 2007b, 2012), Abdala et al. (2012b), Breitman et al. (2011b), Carrasco et al. (2010), Carrera and Avila (2008a, 2008b), Cei (1973a, 1974, 1986, 1993, 2003), Cei and Castro (1973), Cei et al. (2001, 2003), Cei and Scolaro (1980), Cei and Scolaro (1999), Cruz et al. (1999), Daciuk and Miranda (1980), Etheridge and Christie (2003), Gallardo (1960), Giambelluca (1999), Giraudo et al. (2012), Giraudo and Scrocchi (2002), Ibargüengoytía and Schulte II (2001), Kluge (1964), Koslowsky (1898), Lobo (2005), Lobo and Quinteros (2005a, 2005b), Lobo et al. (2010), Montero (1996), Nenda et al. (2007), Schulte II et al. (2004), Scolaro (1976a, 1976b, 1990, 1993, 2005, 2006), Scolaro and Cei (1979, 1997, 2006), Scolaro et al. (2005, 2013), Scolaro and Ibargüengoytía (2007), Scolaro and Pincheira-Donoso (2010), Scott Jr. et al. (2006), Victoriano et al. (2010), Williams (1997), Yoke et al. (2006) and Wallach et al. (2014).

We constructed a hexagonal cell grid (White et al. 1992, White 2000) with each entire perimeter cell having an area of 2,787 km^2^, covering the entire territory of Chubut province. The resulting grid contained 106 hexagons. Hexagons are used rather than squares because they possess greater statistical efficiency (Olea 1984) and are more dynamically adaptable (Yfantis et al. 1987), allowing them to adjust to the boundaries of an irregular perimeter (e.g., Chubut province’s coastline). In this approach with continuous tessellations, hexagons have the advantage over squares in that all six adjacent plots of a plot are equally distant, while squares have four closer and four more distant neighbors (Dengler 2009). This facilitates comparison of different data sets by discretizing a large and continuous area (White 2000). The grid was intersected to fit the shape of Chubut province and to restrict the cells to match the limits of the study area. For this grid, we recorded the number of different localities and species richness for each cell. We analyzed species richness, Shannon-Weaver index, Simpson’s index and Jaccard similarity index for Subantártica, Patagónica and del Monte phytogeographic provinces (Roig 1998) using a shapefile created and provided by the National Environment Secretary (SAyDS 1997). To remove the potential bias of uneven catch rates, rarefaction was used to compare species richness (Gotelli and Colwell 2001, Buddle et al. 2005). We used QUANTUM GIS 2.6® (Open Source Geospatial Foundation Project Development Team 2014) for spatial and species richness analyses and to elaborate species geographic distribution maps. All statistical analyses were performed with R 3.0.2 (R Core Team 2014) and VEGAN PACKAGE 2.0–9 (Oksanen et al. 2013). Additional data taken from the literature were cited literally and only mapped when the data was from vouchered specimens with accurate coordinates or location. The conservation status of each species was taken from Abdala et al. (2012a). Geographic records considered erroneous or outdated were discussed in the taxonomic comments section.

## Results

### Richness and diversity

**Figure 1.**
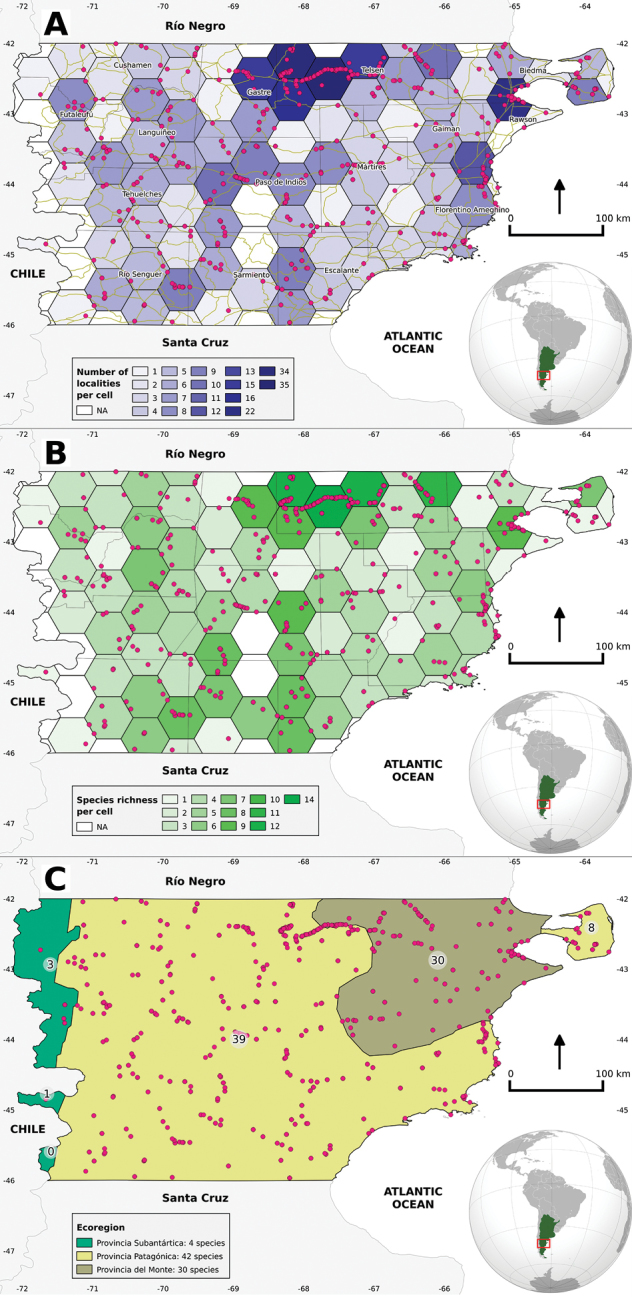
**A** Presence of reptiles recorded for central Patagonia, based on a spatial grid. Blue gradient grid: representing the number of localities sampled within each cell; brown lines: roads from a vector line shapefile; department’s names and main geographic references are presented **B** Species richness of reptiles recorded for central Patagonia, analyzed based on a spatial grid. Green gradient grid: representing the richness within each cell **C** Species richness of reptile recorded for central Patagonia, analyzed based on phytogeographic provinces. White circles: representing the richness within each phytogeographic province polygon; map legend: total species per phytogeographic province. References: magenta dots: localities with accurate location information.

We compiled a total of 2,842 reptile presence records (Fig. 1) distributed over 16 departments, 2,720 correspond to lizards (162 Leiosauridae, 2,302 Liolaemidae, 253 Phyllodactylidae and 3 Teiidae), 107 to snakes (89 Dipsadidae and 18 Viperidae), 14 to amphisbaenians (Amphisbaenidae) and one was a turtle (Cheloniidae) (Tables 1–3). These records represent eight families, 18 genera and 52 reptile species present in Chubut province.

**Table 1.** Presence of reptiles for Chubut province. References: A = LJAMM-CNP, B = museum or literature, C = both. Departments: 1 = Biedma, 2 = Cushamen, 3 = Escalante, 4 = Florentino Ameghino, 5 = Futaleufú, 6 = Gaiman, 7 = Gastre, 8 = Languiñeo, 9 = Mártires, 10 = Paso de Indios, 11 = Rawson, 12 = Río Senguer, 13 = Sarmiento, 14 = Tehuelches, 15 = Telsen, 16 = Without department information, 17 = phytogeographic provinces (PS – Subantártica, PP – Patagónica, PDM – del Monte).

	1	2	3	4	5	6	7	8	9	10	11	12	13	14	15	16	17
AMPHISBAENIDAE																	
*Amphisbaena plumbea* (Fig. 3B)	C			C											A	B	PP, PDM
*Amphisbaena kingii* (Fig. 3B)	B										B						PDM
CHELONIIDAE																	
*Chelonia mydas* (Fig. 3A)	B																PP
DIPSADIDAE																	
*Paraphimophis rustica*	B															B	PP, PDM
*Erythrolamprus sagittifer sagittifer* (Fig. 3A)	B								A						B	B	PDM
*Xenodon semicinctus*																B	
*Oxyrhopus rhombifer* (Fig. 3)	B														B		PDM
*Phalotris bilineatus* (Fig. 3A)	B																PDM
*Philodryas patagoniensis* (Fig. 3)	C			A						A	A				A	B	PP, PDM
*Philodryas psammophidea*	B										B						PDM
*Philodryas trilineata* (Fig. 3A)	C					A			A						A	B	PDM
*Pseudotomodon trigonatus* (Fig. 3A)	C			C						A					A	B	PP, PDM
*Tachymenis chilensis* (Fig. 3A)		B			C											B	PP
LEIOSAURIDAE																	
*Diplolaemus bibronii* (Fig. 3B)	B		C							C		C	C	A	A	B	PP
*Diplolaemus darwinii* (Fig. 3B)			C	C								A				B	PP
*Diplolaemus sexcinctus* (Fig. 3B)		A					A	A							A	B	PP
*Leiosaurus bellii* (Fig. 3B)	C		B	B		A				A	A				A	B	PP, PDM
*Pristidactylus nigroiugulus* (Fig. 3B)			A				A	A		C					C		PP, PDM
PHYLLODACTYLIDAE																	
*Homonota darwinii* (Fig. 3C)	C	A	C	A			A	A	A	A		C	C		A	B	PP, PDM
TEIIDAE																	
*Aurivela longicauda* (Fig. 3B)	C														A		PDM
VIPERIDAE																	
*Bothrops ammodytoides* (Fig. 3A)	B		B												A	B	PP, PDM

**Table 2.** Presence of Liolaemidae taxa for Chubut province. References: A = LJAMM-CNP, B = museum or literature, C = both. Departments: 1 = Biedma, 2 = Cushamen, 3 = Escalante, 4 = Florentino Ameghino, 5 = Futaleufú, 6 = Gaiman, 7 = Gastre, 8 = Languiñeo, 9 = Mártires, 10 = Paso de Indios, 11 = Rawson, 12 = Río Senguer, 13 = Sarmiento, 14 = Tehuelches, 15 = Telsen, 16 = Without department information, 17 = phytogeographic provinces (PS – Subantártica, PP – Patagónica, PDM – del Monte).

	1	2	3	4	5	6	7	8	9	10	11	12	13	14	15	16	17
LIOLAEMIDAE																	
*Liolaemus bibronii* (Fig. 3D)		A	A	C	A		A	A	A	A		A	A	A	A	B	PP, PDM
*Liolaemus boulengeri* (Fig. 3D)	B	C	A	C	B	B	A	C	A	A		C	A	C	A	B	PP, PDM
*Liolaemus camarones* (Fig. 3E)				C													PP
*Liolaemus canqueli* (Fig. 3E)							A	A	A	C						B	PP, PDM
*Liolaemus chehuachekenk* (Fig. 3E)		A					A	A		A					A		PP, PDM
*Liolaemus darwinii* (Fig. 3E)	C					A			A		C				C	B	PP, PDM
*Liolaemus elongatus* (Fig. 3E)		A			A			C		A		A	C	C	A	B	PP, PS
*Liolaemus fitzingerii* (Fig. 3E)	B		C	C						A		A	A	A		B	PP, PDM
*Liolaemus gracilis* (Fig. 3E)	C					A									A	B	PP, PDM
*Liolaemus kingii* (Fig. 3F)		A	C		A			C		C		C	C	A		B	PP, PS
*Liolaemus kriegi* (Fig. 3F)		B														B	PP
*Liolaemus lineomaculatus* (Fig. 3F)			C					C				C		A		B	PP, PS
*Liolaemus morandae* (Fig. 3F)			C									A					PP
*Liolaemus melanops* (Fig. 3F)	C					A			C	A	C				C	B	PP, PDM
*Liolaemus petrophilus* (Fig. 3F)		A					A	A	C	C			A		A		PP, PDM
*Liolaemus pictus argentinus* (Fig. 3G)					A			A								B	PS
*Liolaemus rothi* (Fig. 3G)		A					A								C	B	PP, PDM
*Liolaemus senguer* (Fig. 3G)										A		C		C			PP
*Liolaemus shehuen* (Fig. 3E)															C		PP, PDM
*Liolaemus somuncurae* (Fig. 3G)															A		PP
*Liolaemus telsen* (Fig. 3G)															C		PP, PDM
*Liolaemus uptoni* (Fig. 3G)							C										PP
*Liolaemus xanthoviridis* (Fig. 3G)			A	C		B			A	A	C					B	PP, PDM
*Phymaturus calcogaster* (Fig. 3H)															C		PP, PDM
*Phymaturus camilae* (Fig. 3H)								B									PP
*Phymaturus castillensis* (Fig. 3H)													B				PP
*Phymaturus felixi* (Fig. 3H)										C							PP
*Phymaturus indistinctus* (Fig. 3H)												C	B				PP
*Phymaturus patagonicus* (Fig. 3H)						B				C					C	B	PP, PDM
*Phymaturus somuncurensis* (Fig. 3H)															C	B	PP
*Phymaturus videlai* (Fig. 3H)													B				PP

**Table 3.** Reptile list records based on the information source: A) number of family records from the LJAMM-CNP collection, B) number of family records from literature and museum information, C) number of total records per family, D) number of genera per family, E) number of species per genus.

Families	A (n = 2222)	B (n = 620)	C (n = 2832)	D (n = 18)	E (n = 52)
Amphisbaenidae	4	10	14	1	2
Cheloniidae	0	1	1	1	1
Dipsadidae	35	54	89	8	10
Leiosauridae	96	66	162	3	5
Liolaemidae	1840	462	2302	2	31
Phyllodactylidae	244	9	253	1	1
Teiidae	1	2	3	1	1
Viperidae	2	16	18	1	1

The families that showed the highest species number were Liolaemidae and Dipsadidae with 31 and 10 species respectively (Table 3). Dipsadidae also has the greatest number of genera represented (eight, Table 3). Liolaemidae and Phyllodactylidae were the groups that had the highest number of records with 2,302 and 253 respectively (Table 3). Species number recorded within political Departments varies between six and 27 for Futaleufú and Telsen, respectively (Tables 1–2). The highest number of records were recorded for Telsen (664) and Paso de Indios (410) Departments (Table 4). There are 2,222 LJAMM-CNP collection records for this province; whereas there are 620 literature and museums records, of which 127 do not clearly specify the Department and were not mapped (Table 4).

**Table 4.** Reptile records for political department based on the information source: A) number of family records from the LJAMM-CNP collection, B) number of family records from literature and museum information, C) total records per political department.

Political departments	A (n = 2222)	B (n = 620)	C (n = 2842)	Area km^2^
Biedma	169	63	232	12920.36
Cushamen	76	28	104	16312.96
Escalante	174	13	187	14286.51
Florentino Ameghino	139	48	187	15866.99
Futaleufú	31	12	43	9162.13
Gaiman	28	32	60	11633.59
Gastre	104	11	115	15996.02
Languiñeo	150	36	186	14798.94
Mártires	96	5	101	15645.31
Paso de Indios	326	84	410	22232.58
Rawson	32	17	49	4151.81
Río Senguer	134	29	163	22868.47
Sarmiento	86	26	112	14543.86
Tehuelches	81	21	102	14594.87
Telsen	596	68	664	19459.08
Without department information	0	127	127	----

The cells from central-east of Telsen (e.g. 35 and 34 localities) and west of Gastre (14 localities) Departments and the area around Puerto Madryn city (22 localities), represent the most over-sampled regions of Central Patagonia (Fig. 1A), for which we found higher richness. Although the number of sampled localities in the cells around Puerto Madryn (*S* = 9), Paso de Indios (*S* = 9), Sarmiento (*S* = 8), Río Senguer (*S* = 8) and Escalante (*S* = 8) was only moderate, they also supported a relatively high number of species (Fig. 1B). The Patagónica was the phytogeographic province with the highest species richness (*S* = 42), followed by del Monte (*S* = 30) and Subantártica (*S* = 4; Fig. 1C).

The highest reptile diversity was recorded for the Patagónica province (*H* = 2.98898; *D* = 0.9330269), while the lowest diversity was found for the Subantártica province (*H* = 1.232643; *D* = 0.6632653, Table 5). The most similar phytogeographic provinces in terms of their species’ composition were Patagonian and del Monte Provinces (d_jk_ = 0.8839369), while the Subantártica province shares all its species with the Patagónica province (d_jk_ = 0.9943445), but does not share any species with del Monte province (Table 5). Regarding the exclusive occurrence of species in relation to the boundaries of each phytogeographic province, the Patagónica supports 17 unique species, while del Monte has eight and the Subantártica has only *Liolaemus pictus argentinus* with no records in the other phytogeographic provinces (Table 2). The genus *Diplolaemus* was only present in Patagónica province and del Monte province was represented mostly by snakes (Table 1). Rarefaction estimates of species richness indicated that Patagónica accumulated species faster than did the other phytogeographic provinces (Supplementary file 1: Fig. 1).

**Table 5.** Species diversity in central Patagonia, Argentina: PS) Subantártica province, PP) Patagónica province, PDM) del Monte province.

Diversity	Species richness (*S*)	Shannon-Weaver’s index (*H*)	Simpson’s index (*D*)
PS	4	1.232643	0.6632653
PP	42	2.98898	0.9330269
PDM	30	2.513668	0.8555218
**Jaccard index (d_jk_)**	PS	PP	PDM
PS	0	0.9943445	1
PP	0.9943445	0	0.8839369
PDM	1	0.8839369	0

We recorded five zoogeographical novelties: (1) First record of *Pseudotomodon trigonatus* for Telsen Department; (2) southernmost record of *Liolaemus gracilis* in Argentina and first vouchered presence for Gaiman Department; (3) first records of *Liolaemus kingii* for Cushamen, Escalante, Futaleufú, Languiñeo, Paso de Indios, Río Senguer and Tehuelches Departments; (4) first records of *Liolaemus rothi* for Cushamen and Gastre Departments; (5) first records of *Phymaturus indistinctus* for Río Senguer Department. The reptile species list for Chubut province is detailed in Tables 1 and 2.

### Taxonomic comments

Based on the reptile species list for Chubut province and updated species distribution detailed above; we provide specific comments for published records for which we detected problems:

1. Montero (1996) cited two records of *Amphisbaena kingii* (Bell, 1833) vouchered as CHINM 1759–60, but we did not include them in a map because the author´s coordinates correspond to a location in the sea.
2. We did not consider Dixon and Thomas’s (1982) presence record of *Erythrolamprus sagittifer sagittifer* for Chubut, because these authors did not include either a literature record or vouchered specimens.
3. Giraudo and Scrocchi (2002) cited *Micrurus pyrrhocryptus* (Cope, 1862) for Chubut province. At the present time we cannot confirm the presence of this species in Chubut because no voucher specimens are deposited in a herpetological collection reviewed by us and no bibliographic citation was made based on a particular specimen.
4. We did not take into account the records of *Liolaemus ceii* (Donoso-Barros, 1971) for Nahuel Pan, Futaleufú Department, cited as the southernmost limit of this species by Cei (1986) and Scolaro (2005) for the northwestern area of Chubut, because we could not verify any vouchered specimen from this area.
5. We did not consider the records for *Liolaemus kingii* for Península Valdés (CENAI 1761), *Liolaemus lineomaculatus* (CENAI 1768 = JD-Z 1589) for Puerto Madryn and *Liolaemus melanops* (CENAI 854 = JD-Z 1734) for Sierra Cuadrada from Daciuk and Miranda (1980). Current distribution of *Liolaemus kingii* and *Liolaemus lineomaculatus* is well studied and their range of distribution is much further south than the city of Puerto Madryn (Breitman pers. comm.). This was well analyzed, mapped and verified in Breitman et al. (2011a, 2011b, 2011c, 2012, 2013). The locality in which Daciuk and Miranda (1980) cited a specimen identified as *Liolaemus melanops* was subsequently recognized to harbor populations of *Liolaemus canqueli* (Cei and Scolaro 1980, 1983). We considered these records of *Liolaemus kingii*, *Liolaemus lineomaculatus* and *Liolaemus melanops* as potentially erroneous, based on the taxonomic and distributional updates reviewed and considered in this work.
6. *Liolaemus wiegmannii* (Daciuk and Miranda 1980, Etheridge 2000) is a species cited for Chubut based on specimens purportedly collected in the province but we think this information represents an error at either the taxonomic or geographic level. This record of *Liolaemus wiegmannii* in Bahía del fondo (Chubut province, Etheridge 2000) is separated by approximately 560 km in a straight line from the southernmost locality known in Río Negro province (see review of this species group, Avila et al. 2009). This is the only provincial record for this species and is in complete isolation of populations mentioned above. In addition, we were unable to review this specimen ourselves. We considered that future surveys are needed to conclusively determine its presence in Chubut province.
7. We did not consider the records IBA-UNC N°1142, 1076, 1075 CNP N°28, 33–4, 79 for *Liolaemus goetschi* (Müller and Hellmich 1938) cited by Scolaro (1976b) in Península Valdés. This taxon has been recently redescribed (Nori et al. 2010a) and the current known populations are restricted to the north of Río Negro province (Nori et al. 2010b, Pérez et al. 2011) approximately 430 km in a straight line from Península Valdés. The populations cited as *Liolaemus goetschi* in Scolaro (1976b) are considered as *Liolaemus melanops* since subsequent works showed molecular (Avila et al. 2006b) and morphological (Abdala 2007, Abdala et al. 2012b) differences between these two taxa.
8. We did not include on a map the reference for *Liolaemus lineomaculatus* Boulenger, 1885 MLP.S. 2106 (Ibargüengoytía et al. 2001), located in Escalante Department, because the author´s coordinates correspond to a locality 224 km N (straight line distance) in Mártires Department. This record should be re-examined and compared with new and recently described species (Breitman et al. 2011b) from this group of lizards.
9. We consider that, the taxonomic identity for the records of *Liolaemus xanthoviridis* (Cei and Scolaro 1980) made by Cruz et al. (1999) for Península Valdés should be checked based on updated taxonomic proposals. The populations of Península Valdés considered as *Liolaemus xanthoviridis* by Cruz et al. (1999), have subsequently been considered to be *Liolaemus melanops* based on molecular (Avila et al. 2006b) and morphological (Abdala 2007, Abdala et al. 2012b) differences.

## Discussion

Knowledge about world biodiversity remains inadequate because most species living on Earth are still not formally described (the Linnean shortfall) and because geographical distributions of most species are poorly understood and usually contain many gaps (the Wallacean shortfall; Bini et al. 2006). Regional lists are small steps towards solving some of these problems, and checklists with geographic and voucher information, despite their limitations, are a good start for further detailed studies. As Rivas et al. (2012) state, checklists are dynamic and should be considered as a still frame in time that has no lasting value, only showing the state of knowledge at a particular moment. Reports of new species, synonymizations and elevation of old synonyms to specific status, clarification of prior mistakes and new data about species distributions rapidly change our knowledge of biological diversity. Here we present a comprehensive background useful to other biologists for future, more detailed works. Based on this review, the reptile fauna of central Patagonia is dominated by lizards, both in species diversity and number of records. On the contrary, the regional distribution of snakes are rather marginal and for most of them, this area represents the southernmost limit of their geographic range, since the majority of the species to be related with the del Monte or ecotonal areas with Patagónica province (except *Tachymenis chilensis*).

Some biases are evident in our study; north-central and northeastern areas of the Chubut province have a high number of data because they were more intensively sampled due to their proximity to our research center, or because they were used in several ecological studies and have easy access by road or trails (Fig. 1A). Some areas located far away from our research center need greater sampling effort, such as the central-south and the Subantártica province, where no information is available for some grid cells (Figs 1B, C). Information about reptile distribution from central Patagonia is scarce and access to specimens deposited in public herpetological collections or bibliographic references with accurate locality information is relatively rare. The majority of the species were relatively recently reviewed (see bibliography) and some old taxonomic problems were partially solved (e.g., Abdala 2003, Lobo and Quinteros 2005b, Lobo et al. 2010); but for some species complexes, the taxonomic status of some populations and species limits are still unclear (e.g., Morando et al. 2013). The only turtle cited for central Patagonia is the marine species *Chelonia mydas*, but the cited specimen probably corresponds to a lost individual, since coastal Chubut areas are not in the feeding or nesting activity range of this species (Falabella et al. 2009).

The spatial occurrence of *Homonota darwinii* is fragmented across the studied area with two distributional gaps: a western strip and central and eastern areas of the Chubut province (Fig. 3C). On the other hand, *Liolaemus bibronii* and *Liolaemus boulengeri* were the taxa most evenly distributed along the studied region, although they were presented by few records for the del Monte province (Fig. 3D). Some recently described species (e.g. *Liolaemus camarones*, *Liolaemus shehuen*, Fig. 3E; *Liolaemus morandae*, Fig. 3F; *Liolaemus senguer*, Fig. 3G; *Phymaturus camilae*, *Phymaturus castillensis*, *Phymaturus felixi* and *Phymaturus videlai*, Fig. 3H) need further studies on their geographic distribution, whereas other species previously cited for the province were not found in any of the collections studied or collected / observed in the field, despite being easily detected or sampled in other areas of their distribution (e.g. *Liolaemus somuncurae*, *Liolaemus kriegi*). Some citations for the region were considered here as taxonomic misidentifications, such as *Liolaemus goetschi*, which is restricted to northern Río Negro and southern La Pampa provinces (Nori et al. 2010a, 2010b), whereas other records require new investigation and/or re-examination (e.g. *Liolaemus wiegmannii*, Etheridge 2000).

**Figure 2.**
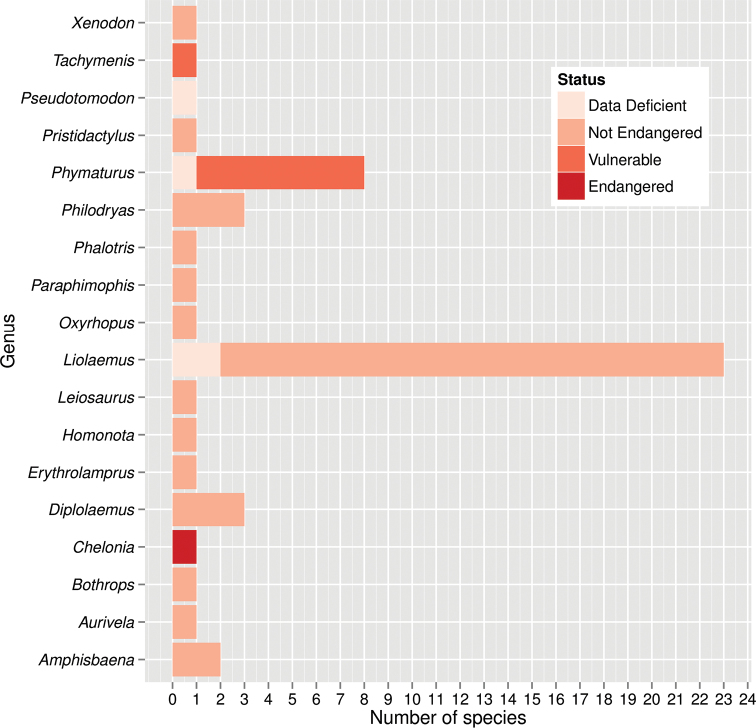
Reptile species conservation status per genus for central Patagonia, Argentina.

The most remarkable results from a conservation status standpoint are that only one taxon (*Chelonia mydas*) is considered endangered, seven of the eight *Phymaturus* species are vulnerable and *Psuedotomodon trigonatus* is data deficient (Fig. 2). The analyses of conservation status by phytogeographic provinces showed that, Patagónica province had the largest number of vulnerable (8) and endangered (1) taxa. Additionally, Subantártica province was the province with the lowest number of taxa (0) with data deficient status, followed by del Monte (1). Our study reveals the small geographic distribution of each of the *Phymaturus* species, of which almost all were recently considered as “vulnerable” (Abdala et al. 2012a). This genus is characterized by living in rocky habitats, exhibiting a high degree of endemism and being viviparous and herbivorous (Abdala et al. 2012a). Thus, we consider that most future management decisions should address the conservation of threatened populations of different *Phymaturus* species.

**Figure 3.**
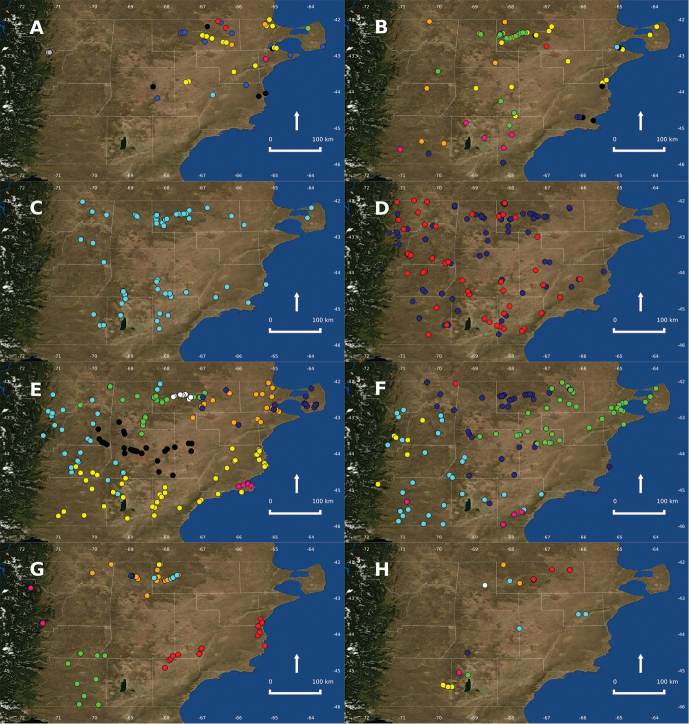
Imagery source: Blue Marble Next Generation (true-color), Web Map Service (WMS) layer from CREAF MAP SERVER (open-gis), EPSG: 4326. **A** Records of Cheloniidae, Dipsadidae and Viperidae. Green dot: *Chelonia mydas*; light blue dot: *Erythrolamprus sagittifer sagittifer*; magenta dot: *Phalotris bilineatus*; red dots: *Bothrops ammodytoides*; orange dots: *Oxyrhopus rhombifer*; black dots: *Pseudotomodon trigonatus*; blue dots: *Philodryas patagoniensis*; yellow dots: *Philodryas trilineata*; grey dots: *Tachymenis chilensis* **B** Records of lizards. Black dots: *Amphisbaena plumbea*; light blue dot: *Amphisbaena kingii*; red dot: *Aurivela longicauda*; magenta dots: *Diplolaemus bibronii*; blue dots: *Diplolaemus darwinii*; orange dots: *Diplolaemus sexcinctus*; yellow dots: *Leiosaurus bellii*; green dots: *Pristidactylus nigroiugulus* **C** Records of *Homonota darwinii* **D** Records of some *Liolaemus* species. Blue dots: *Liolaemus bibronii*; red dots: *Liolaemus boulengeri* **E** Records of some *Liolaemus* species. Magenta dots: *Liolaemus camarones*; black dots: *Liolaemus canqueli*; green dots: *Liolaemus chehuachekenk*; orange dots: *Liolaemus darwinii*; light blue dots: *Liolaemus elongatus*; yellow dots: *Liolaemus fitzingerii*; blue dots: *Liolaemus gracilis*; white dots: *Liolaemus shehuen* **F** Records of some *Liolaemus* species. Light blue dots: *Liolaemus kingii*; red dot: *Liolaemus kriegi*; yellow dots: *Liolaemus lineomaculatus*; green dots: *Liolaemus melanops*; magenta dots: *Liolaemus morandae*; blue dots: *Liolaemus petrophilus* **G** Records of some *Liolaemus* species. Magenta dots: *Liolaemus pictus argentinus*; orange dots: *Liolaemus rothi*; green dots: *Liolaemus senguer*; yellow dot: *Liolaemus somuncurae*; light blue dots: *Liolaemus telsen*; blue dots: *Liolaemus uptoni*; red dots: *Liolaemus xanthoviridis* **H** Records of *Phymaturus* species. Red dots: *Phymaturus calcogaster*; white dot: *Phymaturus camilae*; green dot: *Phymaturus castillensis*; blue dot: *Phymaturus felixi*; yellow dots: *Phymaturus indistinctus*; light blue dots: *Phymaturus patagonicus*; orange dots: *Phymaturus somuncurensis*; magenta dot: *Phymaturus videlai*.

In summary, the systematic knowledge of several groups are essential to conservation decisions (see Cook et al. 2013), especially for the genera *Liolaemus*, *Phymaturus*, *Pristidactylus* and *Diplolaemus*, which require further taxonomic studies. Studies that update and review species’ geographic distribution coupled with their taxonomic status are necessary (Feeley and Silman 2011) as they provide basic information for biogeographic (Corbalán and Debandi 2008), systematic (Debandi et al. 2012), and conservation (Corbalán et al. 2011, Katzner et al. 2011, Böhm et al. 2013) approaches. Numerous records of lizard population extinctions have been reported worldwide (Sinervo et al. 2010), and there is no doubt that the information presented here will be a useful contribution for future analyses of climate driven population extinction, as well as for the development of conservation plans.

## Supplementary materials

Supplementary material 1 Specimens examined from LJAMM-CNP herpetological collection Data type: Portable Document Format (pdf). Explanation note: Specimens examined from LJAMM-CNP herpetological collection, museum voucher and bibliography data for the reptile’s distribution in Chubut province, Patagonia, Argentina. Additional figures of the results section. This dataset is made available under the Open Database License (http://opendatacommons.org/licenses/odbl/1.0/). The Open Database License (ODbL) is a license agreement intended to allow users to freely share, modify, and use this Dataset while maintaining this same freedom for others, provided that the original source and author(s) are credited.Ignacio Minoli, Mariana Morando, Luciano Javier Avila
