# Supplementary material for: Reptiles of Chubut province, Argentina: richness, diversity, conservation status and geographic distribution maps
Source: Zookeys. 2015 Apr 21;(498):103–26. doi: 10.3897/zookeys.498.7476 (PMC4410151; doi:10.3897/zookeys.498.7476)
Supplement: Supplementary material 1 — Specimens examined from LJAMM-CNP herpetological collection [file zookeys-498-103-s001.pdf]

Reptiles of Chubut Province, Argentina: richness, diversity, conservation status and geographic distribution maps

by

Ignacio Minoli, Mariana Morando & Luciano Javier Avila

SUPPLEMENTARY FILE 1

Specimens examined

Testudines Linnaeus, 1758
Cheloniidae Oppel, 1811

Chelonia mydas (LINNAEUS, 1758)
Reference material (Fig. 6):
Literature and museum citations:
Biedma Department: Scolaro (1990): CNP-H #0331, near Puerto Madryn (42°15'S, 64°04'W).
Conservation status and phytogeographic provinces: endangered, Provincia Patagónica.

Serpentes Linnaeus, 1758
Dipsadidae Bonaparte, 1838

Pseudotomodon trigonatus (LEYBOLD, 1873)
Reference material (Fig. 6):
Biedma Department: LJAMM-CNP 6479, Puerto Madryn (42°48'47.6"S, 65°02'48"W); LJAMM-CNP 14356, Quintas del Mirador, Puerto Madryn (42°48'37.6"S, 65°02'34"W).
Florentino Ameghino Department: LJAMM-CNP 4947, Punta Tombo Natural Protected Area border (44°02'57.2"S, 65°14'31.4"W).
Paso de Indios Department: LJAMM-CNP 11082, National Road 25, 10 km E Los Altares (Los Altares Valley) (43°51'54.0"S, 68°21'26.4"W; Avila 2009b).
Telsen Department: LJAMM-CNP 13239, Road between Cona Niyeu and Telsen, 40.4 km junction Provincial Road 8 (42°12'10.2"S, 66°51'22.6"W).
Literature and museum citations:
Florentino Ameghino Department: Cruz et al. (1999): UNMDP 524, one specimen for Cantera Las Lajas, 20 km S Punta Tombo, 1 km E Provincial Road 1 (44°07'57"S, 65°26'W).
Biedma Department: Daciuk & Miranda (1980): CENAI 3866 (JD-Z 1335), for Carlos Ameghino isthmus, Península Valdés; CENAI 3867 (JD-Z 1336), south shore of San José Gulf, Península Valdés; JD-Z 1640, Punta Cuevas, Puerto Madryn. Scolaro & Cei (1979): confirmed record for Puerto Madryn. Cited for ¨Chubut¨ by Cei (1986) and Giraudo & Scrocchi (2002), without department. Cited for NE corner of Chubut by Scolaro (2005).
Conservation status and phytogeographic provinces: data deficient, Provincia Patagónica and Provincia del Monte.

Erythrolamprus sagittifer sagittifer (JAN, 1863)
Reference material (Fig. 6):

Florentino Ameghino Department: MACN 39041, Chico River, S Florentino Ameghino dam (44°05'31"S, 66°42'18"W; Avila 2009a).
Literature and museum citations:
Cited for proximities of Puerto Madryn and Telsen by Cei (1986). Cited without department by Giraudo & Scrocchi (2002), and for N of Chubut River by Scolaro (2005) for an area including N of Chubut River.
Conservation status and phytogeographic provinces: not endangered, Provincia del Monte.

Xenodon semicinctus (DUMÉRIL, BIBRON & DUMÉRIL, 1854)
Literature and museum citations:
Cited without department by Giambelluca 1999, Giraudo & Scrocchi (2002), (Nenda et al. 2007) and (Wallach et al. 2014).
Conservation status: not endangered.

***Phalotris bilineatus* (**DUMÉRIL, BIBRON & DUMÉRIL, 1854)
Reference material (Fig. 6):
Literature and museum citations:
Rawson Department: Avila et al. (2001): FML 9324, National Road 3, 17 km N Trelew (43°05'52"S, 65°14'17"W).
Biedma Department: Scolaro & Cei (1979): IBA-UNC 1225–1, 2, 3, nearby Puerto Madryn.
Conservation status and phytogeographic provinces: not endangered, Provincia del Monte.

Philodryas patagoniensis (GIRARD, 1858)
Reference material (Fig. 6):
Biedma Department: LJAMM-CNP 4948, Puerto Madryn (42°44'51.3"S, 65°05'29.8"W); LJAMM-CNP 4500, road to Punta Delgada, Península Valdés (42°45'18.39"S, 63°39'47.41"W).
Florentino Ameghino Department: LJAMM-CNP 6117, Provincial Road 3, 80 km S Trelew (43°49'08.8"S, 65°47'12.3"W).
Paso de Indios Department: LJAMM-CNP 3861, Provincial Road 27, 4 km S El Sombrero and junction Provincial Road 53 (44°10'60.0"S, 68°14'40"W).
Rawson Department: LJAMM-CNP 6763, Provincial Road 5, to Bahía Cracker, 2.5 km access to Urtazúm Hermanos Ranch (42°59'08.7"S, 64°33'28.4"W).
Telsen Department: LJAMM-CNP 6027, Provincial Road 4, 62.7 km W Telsen (42°22'40.0"S, 67°35'21"W); LJAMM-CNP 3241, Provincial Road 4, Mallín Grande Ranch Post (42°22'14.9"S, 67°31'11.1"W); LJAMM-CNP 3428, Provincial Road 4, 3.5 km E Mallín Grande Ranch (42°21'47.8"S, 67°28'54.3"W); LJAMM-CNP 11199, Provincial Road 8, 102 km NW junction Provincial Road 4 (42°05'32.6"S, 66°29'02.5"W); LJAMM-CNP 11200, Provincial Road 8, 48 km NW junction Provincial Road 4, El Moro Ranch (42°23'06.1"S, 66°10'19.7"W); LJAMM-CNP 3682, Provincial Road 4, 56.4 km E Telsen (42°37'46.9"S, 66°55'38.4"W).
Literature and museum citations:
Biedma Department: Citations by Daciuk & Miranda (1980): CENAI 3861 (JD-Z 1327), CENAI 3870 (JD-Z 345), 3873 (JD-Z 1330), south shore San José Gulf, Península Valdés; JD-Z 1328, Carlos Ameghino isthmus, La Isla ranch, Península Valdés; CENAI 3872 (JD-Z 1329), Carlos Ameghino isthmus, Península Valdés; CENAI 3874 (JD-Z 1334) La Isla Ranch, south shore San José Gulf.
Giraudo & Scrocchi (2002): cited without department. Scolaro (2005): cited for center and NE of Chubut.
Conservation status and phytogeographic provinces: not endangered, Provincia Patagónica and Provincia del Monte.

Philodryas trilineata (BURMEISTER, 1861)
Reference material (Fig. 6):
Biedma Department: MLP.R 5064, National Road 3, 68.4 km S Sierra Grande, 23 km S Arroyo Verde (42°10'56.3"S, 65°13'25.9"W); LJAMM-CNP 13654, Provincial Road 1, 48 km N from junction Provincial Road 2 (42°11'35.6"S, 65°03'05.1"W); LJAMM-CNP 8236, S Puerto Madryn (42°47'22"S, 65°00'17.9"W); LJAMM-CNP 8237, Playa Paraná near Puerto Madryn (42°47'48.4"S, 64°56'34.6"W); LJAMM-CNP 14417, Puerto Madryn (42°47'04.7"S, 65°00'29.1"W).
Gaiman Department: LJAMM-CNP 6116, National Road 25, 40 km SW Dolavon (43°27'15.8"S, 66°07'16.3"W); LJAMM-CNP 6118, National Road 25, 1 km W Gaiman entrance (43°16'55.3"S, 65°31'47.7"W).
Mártires Department: LJAMM-CNP 6480, National Road 25, 281 km Puerto Madryn, 9 km W Las Plumas (43°43'15.05"S, 67°22'32.53"W); LJAMM-CNP 6483, National Road 25, 281 km Puerto Madryn, 10 km W Las Plumas (43°44'25.9"S, 67°26'58.9"W).
Telsen Department: LJAMM-CNP 6934, Provincial Road 4, 7 km E Telsen (42°28'25.9"S, 66°52'33.8"W); LJAMM-CNP 7607, Provincial Road 4, 50 km E Telsen (42°37'51.7"S, 66°25'25.3"W); LJAMM-CNP 10481, Provincial Road 4, 38 km W Telsen (42°26'42.1"S, 66°59'33.5"W); LJAMM-CNP 11048, Provincial Road 4, 86 km W Sierra Chata, 8 km E Telsen (42°28'25.6"S, 66°52'32.3"W); LJAMM-CNP 11127, Provincial Road 4. 2 km E Provincial Road 61 (42°31'46.8"S, 66°43'40.2"W); LJAMM-CNP 13222–3, Provincial Road 8, 82.6 km junction Provincial Road 4 (42°14'54.5"S, 66°20'00.9'W); LJAMM-CNP 13854, Provincial Road 4, 31.7 km W Sierra Chata (42°38'36.1"S, 66°19'46.5"W).
Literature and museum citations:
Biedma Department: Cited by Daciuk & Miranda (1980): CENAI 2561 (JD-Z 1712) and CENAI 2562 (JD-Z 1713) Punta Loma, south shore San José Gulf; JD-Z 347, south shore San José Gulf, Península Valdés; CENAI 3868 (JD-Z 1332), Carlos Ameghino isthmus, Península Valdés; CENAI 3869; (JD-Z 1591) Puerto Madryn; (JD-Z 1333), Punta Delgada, Península Valdés. Specimen examined: FML 09206, 5 km W Puerto Lobos, Biedma Department (42°01'07"S, 65°06'15"W).
Cited by Giraudo & Scrocchi (2002) without department and Scolaro (2005) mention the species for the NE of Chubut.
Conservation status and phytogeographic provinces: not endangered, Provincia del Monte.

Philodryas psammophidea GÜNTHER, 1872
Literature and museum citations:
Cited for Biedma and Rawson Departments by Scolaro (2006).
Conservation status and phytogeographic provinces: not endangered, Provincia del Monte.

Paraphimophis rustica (COPE, 1878)
Reference material:
Literature and museum citations:
Biedma Department: Cited by Daciuk & Miranda (1980): JD-Z 346, south shore San José Gulf, Península Valdés. Cited for Península Valdés by Cei (1993).
Cited by Scolaro (2005) for Florentino Ameghino dam. Scott Jr. et al. (2006) made citations for: MACN 5147 (Chubut); CENAI 3875, for Península Valdés, Carlos Ameghino isthmus (= JD-Z 1331; Daciuk & Miranda, 1980).
Conservation status and phytogeographic provinces: not endangered, Provincia Patagónica and Provincia del Monte.

Oxyrhopus rhombifer DUMÉRIL, BIBRON & DUMÉRIL, 1854
Reference material (Fig. 6):

Literature and museum citations:
Biedma Department: Carrera & Avila (2008b): MLP.S 2597, National Road 3, 9 km S junction Provincial Road 60.
Telsen Department: Carrera & Avila (2008a): MACN 39042, María de las Nieves Ranch, on Provincial Road 4, 15–20 km NW Sierra Chata (42.53124°S, 65.6369°W; WGS84).
Conservation status and phytogeographic provinces: not endangered, Provincia del Monte.

Tachymenis chilensis (SCHLEGEL, 1837)
Reference material (Fig. 6):
Futaleufú Department: LJAMM-CNP 14448, El Pinar Ranch (42°55'07.8"S, 71°20'30.9"W; cited by Avila et al., (2012).
Literature and museum citations:
Giraudo et al. (2012) a revision of the geographic distribution of the species and cited 17 observed specimens for Cushamen Department and one vouchered specimen (CENAI 3448) for Futaleufú Department. Giraudo & Scrocchi (2002) mention the species for Chubut without specific locality.
Conservation status and phytogeographic provinces: vulnerable, Provincia Patagónica.

Viperidae Bonaparte, 1831

Bothrops ammodytoides (LEYBOLD, 1873)
Reference material (Fig. 6):
Telsen Department: LJAMM-CNP 3280, Provincial Road 8, 10.5 km S Río Negro and Chubut provincial boundary (42°03'12"S, 66°33'28"W); LJAMM-CNP 13221, Provincial Road 8, 82.6 km junction Provincial Road 4 (42°14'10.6"S, 66°21'00.6"W).
Literature and museum citations:
Biedma Department: Daciuk & Miranda (1980): JD-Z 348a, Punta Norte (La Ernestina Ranch), Península Valdés; CENAI 945–8, National Road 3 and Puerto Lobos road; CENAI 1749 (JD-Z 348b), Punta Norte, La Ernestina Ranch, Península Valdés; CENAI 1750 (JD-Z 349) south shore San José Gulf, Península Valdés; CENAI 1751 (JDZ 1337) stream in San José Gulf; CENAI 1752 (JD-Z 1338) caleta Valdés, Península Valdés; CENAI 1753 (JD-Z 1339) Península Valdés, between Punta Cantor and Punta Hércules; CENAI 1754 (OD-Z 1340) Punta Delgada, Península Valdés; CENAI 1755 (JD-Z 1341) Punta Norte (provincial reserve), Península Valdés; CENAI 1756 (JD-Z 1342), Punta Pirámides, Península Valdés; CENAI 1757 (JD-Z 1349), Punta Pirámides, Península Valdés; CENAI 1758 (JD-Z 1586) shore in front of Quintano island, San Jorge Gulf; JD-Z 344, La Armonía Ranch, Península Valdés; CENAI 3439, Salinas Grandes, Península Valdés; CENAI 3865 (JD-Z 1585) Bajo El Gualicho Ranch, Península Valdés; CENAI 3521, Península Valdés. Carrasco et al. (2010): MLP-JW 563, MACN 29101, MACN 32887–93, MACN 35351, Península de Valdés; MLP-JW 562, Puerto Madryn; MLP-JW 636, Puerto Pirámide; MACN 32293, unknown locality.
Escalante Department: Daciuk & Miranda (1980): CENAI 2939 and CENAI 2956, Comodoro Rivadavia.
Cei (1986): cited for the entire province except for the N-S narrow strip along the Andes Mountains. Giraudo & Scrocchi (2002): cited without department. Scolaro (2005): cited for the entire province. Scolaro (2006): cited for the entire province except for the N-S narrow strip along the Andes Mountains.
Conservation status and phytogeographic provinces: not endangered, Provincia Patagónica and Provincia del Monte.

Squamata Merrem, 1820
Amphisbaenidae Gray, 1865

Amphisbaena plumbea GRAY, 1872
Reference material (Fig. 7):
Biedma Department: LJAMM-CNP 13980, Puerto Madryn (42°47'10.4'S, 64°59'57.0"W).
Florentino Ameghino Department: LJAMM-CNP 11073, Provincial Road 30, 47 km E junction National Road 3 (44°43'00.7"S, 65°60'38.9"W); LJAMM-CNP 14418, Provincial Road 1, 11 km N Reserva Provincial Punta Tombo (43°51'05.6"S, 65°27'24.1"W).
Telsen Department: FML 18118, Road to Laguna de Vaca, 5 km SW Provincial Road 4 (42°26'52.6"S, 67°18'51.6"W; Avila et al. 2007b).
Literature and museum citations:
Biedma Department: Montero (1996): cited for Puerto Madryn, FML 2741, 1BA.UNC 389, MACN 33889 (42°46'S, 65°03'W). Cei (1986): cited for Biedma Department, for southern Chubut up to 45°S and for Chubut and Río Negro provinces boundary. Scolaro (2005): cited for N and NE of Chubut.
Florentino Ameghino Department: Montero (1996): NMBA 6268–270, cited for Camarones (44°48'S, 65°42'W).
Conservation status and phytogeographic provinces: not endangered, Provincia Patagónica and Provincia del Monte.

Amphisbaena kingii (BELL, 1833)
Reference material (Fig. 7):
Literature and museum citations:
Biedma Department: Montero (1996): CHINM 1759–60, South shore San José Gulf, Península Valdés (42°20'S, 64°18'W); MACN 33890–1, Puerto Madryn (42°46'S, 65°03'W). Daciuk & Miranda (1980): CENAI 1759 (JD-Z 1346) south shore of San José Gulf; CENAI 1760 (JD-Z 1348) Punta Gales or Barranca, shore of San José Gulf; JD-Z 1347 Carlos Ameghino isthmus, Península Valdés. Scolaro (2005): cited for Biedma Department and partially Rawson Department.
Conservation status and phytogeographic provinces: not endangered, Provincia del Monte.

Teiidae Gray, 1827

Aurivela longicauda (BELL, 1843)
Reference material (Fig. 7):
Telsen Department: MLP.S 2584, Provincial Road 61, 40.3 km junction Provincial Road 11, between Ranquilhuao Ranch and San Manuel Ranch (42°44'48"S, 66°59'54"W; Frutos et al. 2005).
Literature and museum citations:
Scolaro (Scolaro 2005) and Yoke et al. (2006): cited for Biedma Department.
Conservation status and phytogeographic provinces: not endangered, Provincia del Monte.

Leiosauridae Frost et al., 2001

Diplolaemus bibronii BELL, 1843
Reference material (Fig. 7):
Escalante Department: LJAMM-CNP 10203, Holdich Station (45°58'00.1"S, 68°11'58.8"W); LJAMM-CNP 10211–2, Provincial Road 26, 21.1 km NW junction Provincial Road 37, 29.1 km NW junction National Road 26, 3.9 km SW Rancho Grande Ranch (45°33'29.6"S, 68°11'08.9"W); LJAMM-CNP 10295, Provincial Road 25, 3 km SW junction Provincial Road 27, 13 km NE junction Sierra Chaira road (45°11'34.1"S, 67°57'42.2"W).
Paso de Indios Department: LJAMM-CNP 3895–6, Provincial Road 27, 78.1 km S El Sombrero and Provincial Road 53 (44°35'20.3"S, 67°53'47.2"W).
Río Senguer Department: LJAMM-CNP 6503, Provincial Road 51, 2.8 km S Ricardo Rojas

(45°35'53.2"S, 71°02'54"W).
Sarmiento Department: LJAMM-CNP 3847–3999, Provincial Road 26, 52.3 km W junction Provincial Road 25, Pampa de los Guanacos (45°16'43.9"S, 68°43'03.3"W); LJAMM-CNP 3899, Provincial Road 24, 59.2 km N junction Provincial Road 23, in front of Laguna Seca (44°51'57.5"S, 69°13'29.8"W).
Literature and museum citations:
Biedma Department: Daciuk & Miranda (1980): CENAI 1747 (JD-Z 1699), shore in front of Quintano Island, San Jorge Gulf.
Paso de Indios Department: Victoriano et al. (2010): IBA921, adult female, Meseta Canquel; IBA8111, 8112, adult females, Meseta Canquel; IBA8113, juvenile, Meseta Canquel; IBA8011, adult male, road between Paso de Indios and El Sombrero; IBA8012, adult female, road between Paso de Indios and El Sombrero; BA8013, juvenile, road between Paso de Indios and El Sombrero.
Escalante Department: Victoriano et al. (2010): IBA619, adult male, Valle Hermoso; MZUC-8968, adult female, Comodoro Rivadavia.
Río Senguer Department: Victoriano et al. (2010): MZUC-23203, adult male, Río Mayo; MZUC-23328 - 23330, adult males, road between Perito Moreno and Río Mayo.
Sarmiento Department: Specimens examined: MCN 1321, 1323, Sierra de Castillo 27 km junction between Roads 24 and 25, to Buen Pasto.
Specimen examined: IBA909, adult male, Sierra Castillo; IBA838, adult male, road between Las Horquetas and El Sombrero; IBA915, adult male, road between Las Horquetas and El Sombrero; IBA922, adult female, Manantiales; IBA910, adult male, Laguna Payahile.
Cei et al. (2003) and Scolaro (2005): cited for central and southern Chubut.
Conservation status and phytogeographic provinces: not endangered, Provincia Patagónica.

Diplolaemus darwinii BELL, 1843
Reference material (Fig. 7):
Escalante Department: LJAMM-CNP 9204, Holdich Station (45°57'59.9"S, 68°11'58.5"W); LJAMM-CNP 11422 Holdich Station (45°57'58.4"S, 68°12'00.4"W).
Florentino Ameghino Department: LJAMM-CNP 2115, Provincial Road 30, 37 km W Camarones (44°42'05.0"S, 66°08'54"W).
Río Senguer Department: LJAMM-CNP 13069, National Road 40, 2 km S Río Mayo (45°42'57.4"S, 70°15'53"W).
Literature and museum citations:
Escalante Department: Ibargüengoytía & Schulte II (2001): CH 25, for Road 37, 26 km junction roads 37 and 3 (45°41'S, 67°56'W).
Florentino Ameghino Department: Specimens examined: FML 09143 Provincial Road 30, 37 km W Camarones (44°42'05"S, 66°08'54"W).
Cited for southern, central (Scolaro 2005) and northern Chubut (Cei et al. 2003).
Conservation status and phytogeographic provinces: not endangered, Provincia Patagónica.

Diplolaemus sexcinctus CEI, SCOLARO & VIDELA, 2003
Reference material (Fig. 7):
Cushamen Department: LJAMM-CNP 6270, Provincial Road 4, 15.6 km SE Río Chico, across Bajada del Platero (42°03'46.6"S, 70°14'24"W).
Gastre Department: LJAMM-CNP 5959, Provincial Road 59, 24 km SE El Escorial (43°12'22.7"S, 68°25'01.9"W); LJAMM-CNP 6085, Cerro Baritina area, 3 km S Provincial Road 4, 40 km W Gan Gan (42°25'41.8"S, 68°47'49.1"W); LJAMM-CNP 6086, Mallín area nearby Santana Post, 3 km S Provincial Road 4, 40 km W Gan Gan (42°23'51.2"S, 68°51'22.9"W); LJAMM-CNP 6090, Cerro Navidad area, 3 km S Provincial Road 4, 40 km W Gan Gan (42°24'50.8"S, 68°49'17.5"W).
Languiñeo Department: LJAMM-CNP 13082, 6 Hermanos Ranch, 10 km N-NE junction National

Road 25, to Provincial Road 62, Pocitos de Quichaura (43°26'23.9"S, 70°00'12.7"W).
Río Senguer Department: LJAMM-CNP 2127, Provincial Road 20, 4 km N junction Provincial Road 22 (45°25'54.0"S, 69°50'25"W); LJAMM-CNP 13079, National Road 40, 26 km N La Laurita Ranch (44°43'51.7"S, 70°01'34.0"W).
Tehuelches Department: LJAMM-CNP 11616, Provincial Road 63, 20.9 km NE José de San Martín (43°54'34.5"S, 70°18'49.2"W).
Telsen Department: LJAMM-CNP 3443, Provincial Road 67, 31.4 km N Gan Gan (El Lloradero) (42°22'57.9"S, 68°10'45.4"W); LJAMM-CNP 3444, Provincial Road 67, 27 km N Gan Gan (42°24'04.4"S, 68°11'24.8"W); LJAMM-CNP 3445, Provincial Road 67, 30.3 km N Gan Gan (Aguadas) (42°23'26.3"S, 68°12'22.1"W); LJAMM-CNP 3838, Provincial Road 4, 32.8 km E Gan Gan (42°30'07.6"S, 67°55'38.4"W); LJAMM-CNP 5446, Provincial Road 4, 45 km E Gan Gan (42°25'29.2"S, 67°46'49.1"W); LJAMM-CNP 5606, Provincial Road 4, 85 km W Telsen (42°28'05.6"S, 67°51'11.9"W); LJAMM-CNP 5670, 18 km E Gan Gan (42°31'37.1"S, 68°06'19.6"W); LJAMM-CNP 5924, Provincial Road 67, 80.8 km Gan Gan (42°04'29.8"S, 68°09'38.7"W); LJAMM-CNP 6211, Provincial Road 67, 82 km N Gan Gan (42°04'28.8"S, 68°09'13.4"W); LJAMM-CNP 10589, Provincial Road 4, 76.4 km W Gan Gan (42°24'30.0"S, 67°45'09.5"W); LJAMM-CNP 10973, Provincial Road 67, 11.2 km S Río Negro and Chubut provinces boundary (42°04'33.50"S, 68°09'50.36"W).
Literature and museum citations:
Cei et al. (2003): cited for central-south and northeastern areas. Scolaro (2005): cited for central and northwestern areas of Chubut.
Conservation status and phytogeographic provinces: not endangered, Provincia Patagónica.

Leiosaurus bellii DUMÉRIL & BIBRON, 1837
Reference material (Fig. 7):
Biedma Department: LJAMM-CNP 3303, El Centro Ranch, Península Valdés (42°12'18.33"S, 63°56'44.65"W); LJAMM-CNP 6522–6751, Cerro Avanzado (42°50'05.90"S, 64°53'31.10"W); LJAMM-CNP 6894, Puerto Madryn (42°47'29.10"S, 65°02'12.82"W); LJAMM-CNP 13015–6, Cerro Avanzado (42°49'57.25"S, 64°52'58.55"W); LJAMM-CNP 13008, Playa Fracasso, Península Valdés (42°25'45.65"S, 64°07'51.45"W).
Gaiman Department: LJAMM-CNP 2147 Provincial Road 1, 18 km NW Dos Pozos (43°45'21"S, 65°26'59"W); LJAMM-CNP 13667 Provincial Road 40, 57.5 km E junction Provincial Road 11 (43°09'41"S, 66°24'13.7"W).
Paso de Indios Department: LJAMM-CNP 3746 Provincial Road 27, 14.2 km S El Sombrero and Provincial Road 53 (44°16'12.9"S, 68°16'22.9"W); LJAMM-CNP 8835 Provincial Road 40, 15 km NE junction Provincial Road 12 (43°52'26.2"S, 67°58'03.9"W); LJAMM-CNP 10269, 8131 Provincial Road 27, 67.9 km N junction Provincial Road 25, between Meseta Cuadrada and Sierra Cuadrada, 7.9 km S junction Provincial Road 29 (44°40'38.7"S, 67°51'44.2"W); LJAMM-CNP 12188 Provincial Road 53, 9 km SE Paso de Indios (43°54'23.4"S, 68°57'00.6"W).
Rawson Department: LJAMM-CNP 2243–4, 2244, 2453, 2498–9, FML 09463–4 Isla Escondida Bay (43°40'55"S 65°20'23"W).
Telsen Department: LJAMM-CNP 6091 Provincial Road 4, 53 km W Telsen (42°22'05.7"S, 67°32'02.1"W); LJAMM-CNP 6151–2 Provincial Road 67. 40.3 km junction Provincial Road 11, between Ranquilhuao Ranch and San Manuel Ranch (42°38'48.4"S, 68°14'38"W); LJAMM-CNP 8141, Laguna de Vaca road, detour from Provincial Road 4 (42°26'22.5"S, 67°17'13.8"W); LJAMM-CNP 13234, Road between Cona Niyeu and Telsen, 40.4 km junction Provincial Road 8 (42°12'10.2"S, 66°51'22.6"W); LJAMM-CNP 13257, Road between Cona Niyeu and Telsen, 58.6 km junction Provincial Road 8 (42°19'38.2"S, 66°55'45.2"W).
Literature and museum citations:

Biedma Department: Gallardo (1960): MACN 8366, 5142, 7718, 4093, 937–40, 8978, for Puerto Madryn. Scolaro (1976b): CNP 07, for Ameghino isthmus; CNP 22 Isla de los Pájaros. Daciuk and Miranda (1980): JD-Z 1643, south shore of San José Gulf, Península Valdés; CENAI 597, Puerto Madryn; CENAI 788–789–791, Puerto Madryn; CENAI 1710–1, Puerto Madryn; CENAI 1748 (JD-Z 1695), Nuevo Gulf shore, between Puerto Madryn and Punta Cuevas.
Escalante Department: Gallardo (1960): for localities near Comodoro Rivadavia (MACN 9035, 2108, 8701).
Cei (1986) cited this species for localities close to the sea shore, with records for Biedma, Florentino Ameghino and Escalante Departments. Cited by Scolaro (2005) for almost the entire province.
Conservation status and phytogeographic provinces: not endangered, Provincia Patagónica and Provincia del Monte.

Pristidactylus nigroiugulus CEI, SCOLARO & VIDELA, 2001
Reference material (Fig. 7):
Escalante Department**:** LJAMM-CNP 3888, Provincial Road 27, 46.5 km S junction Provincial Road 29 to Garayalde (44°56'07.6"S, 68°01'37"W; Minoli & Avila 2011b).
Gastre Department**:** LJAMM-CNP 6045, Aguada Oveja Muerta area, 3 km S Provincial Road 4, 40 km W Gan Gan (42°26'24.6"S, 68°48'20"W); LJAMM-CNP 6104–5, Cerro Navidad area, 3 km S Provincial Road 4, 40 km W Gan Gan (42°24'50.8"S, 68°49'17.5"W).
Languiñeo Department**:** LJAMM-CNP 13090, 6 Hermanos Ranch, 10 km N-NE junction National Road 25, to Provincial Road 62, Pocitos de Quichaura (43°26'23.9"S, 70°00'12.7"W; Minoli & Avila 2011b).
Paso de Indios Department**:** LJAMM-CNP 3897, Provincial Road 27, 14.2 km S El Sombrero and Provincial Road 53 (44°15'33.8"S, 68°15'34.4"W); LJAMM-CNP 3900–4, Provincial Road 27, 78.1 km S El Sombrero and Provincial Road 53 (44°35'20.3"S, 67°53'47.2"W; Minoli & Avila 2011b).
Telsen Department**:** LJAMM-CNP 3406, Provincial Road 67, 7.9 km N Gan Gan, detour De Mi Car Ranch (42°27'12.5"S, 68°18'49.7"W); LJAMM-CNP 3409, Provincial Road 67, 17.7 km N Gan Gan (2 km detour to Cañada Leona) (42°24'18.8"S, 68°15'27.1"W); LJAMM-CNP 3417, Provincial Road 67, N Gan Gan (42°25'43.6"S, 68°18'11.5"W); LJAMM-CNP 3839, Provincial Road 67, 19.7 km N Gan Gan (42°25'01.8"S, 68°14'57.7"W); LJAMM-CNP 3840–3, 10 km N Gan Gan, entering the first turning off detour to Provincial Road 67 (42°26'44.9"S, 68°18'42.6"W); LJAMM-CNP 5505, Provincial Road 4, 60.4 km W Telsen (42°22'35.4"S, 67°35'42.9"W); LJAMM-CNP 5508, Provincial Road 4, 2 km E Gan Gan (42°31'44.3"S, 68°01'06.6"W); LJAMM-CNP 5603–5, Provincial Road 4, 85 km W Telsen (42°28'05.6"S, 67°51'11.9"W); LJAMM-CNP 5638–9, Provincial Road 4, 70.7 km W Telsen (42°22'55.3"S, 67°42'44.8"W); LJAMM-CNP 5666, Provincial Road 4, 65.5 km W Telsen (42°22'03.8"S, 67°39'22"W); LJAMM-CNP 5669, 15.5 km E Gan Gan (42°31'37.1"S, 68°06'19.6"W); LJAMM-CNP 6254, Provincial Road 67, 21.6 km N Gan Gan (42°25'16.2"S, 68°16'37.4"W); LJAMM-CNP 6757, Provincial Road 67, 15.1 km N Gan Gan (42°25'24.7"S, 68°17'15.8"W); LJAMM-CNP 6760–1, Provincial Road 67, 10 km N Gan Gan (42°27'24.7"S, 68°18'50.3"W); LJAMM-CNP 6903–4, Provincial Road 67, 16 km N Gan Gan (42°25'27.4"S, 68°17'08.8"W).
Literature and museum citations:
Telsen Department: Avila et al. (2003) and Cei (2003): cited for Telsen. Cei et al. (2001) cited for Sierra Negra Plateau: MACN 37092–3; JMC-DC 1197; MCZ R182882, R182883; IBA-UNC R1477; CH-IADIZA 288, 290; JMC-DC 1196; JAS-DC 594.
Paso de Indios Department: Cei et al. (2001): IBA-UNC 784 Sombrero - Paso de Indios Road; IBA-UNC 934, Callejas Post, Canquel Plateau; MHNG 2146–39, 2146–40, Paso de Indios, S of Chubut River.
Conservation status and phytogeographic provinces: not endangered, Provincia Patagónica and Provincia del Monte.

Phyllodactylidae Gamble et al., 2008

Homonota darwinii darwinii BOULENGER, 1885
Reference material (Fig. 8):
Biedma Department: LJAMM-CNP 5966–9, Península Valdés. La Anita Ranch (42°28'57.0"S, 64°05'11"W); LJAMM-CNP 7518, Península Valdés, El Centro Ranch (42°12'13.0"S, 63°58'33"W); LJAMM-CNP 13210, Provincial Road 8, 11.7 km NW junction Provincial Road 4 (42°40'40.1"S, 65°50'03.6"W).
Cushamen Department: LJAMM-CNP 6974–8 Provincial Road 4, 8.4 km SE Chico River, Bajada del Platero (42°02'04"S, 70°18'31.5"W); LJAMM-CNP 6979–80, Provincial Road 4, 56.3 km SE Chico River, 1 km N junction Provincial Road to Fofo Cahuel (42°18'39.7"S, 70°02'12.2"W); LJAMM-CNP 11089, Provincial Road 13, 51.6 km N Paso del Sapo, 1 km N El Portezuelo, to El Molle (42°23'58.7"S, 69°38'46.1"W).
Escalante Department: LJAMM-CNP 3862–3871, Junction Provincial Road 27 and Provincial Road 25 (45°11'35.2"S, 67°56'38.8"W); LJAMM-CNP 9910–6, Provincial Road 25, 20.3 km NW junction Provincial Road 26, La Oración post (45°20'50.3"S, 68°14'43.8"W); LJAMM-CNP 10207–10, Provincial Road 26, 21.1 km NW junction Provincial Road 37, 29.1 km NW junction National Road 26, 3.9 km SW Rancho Grande Ranch (45°33'29.6"S, 68°11'08.9"W); LJAMM-CNP 10265–6, Pampa Pelada, 8.1 km junction Provincial Road 25, to Sierra Chaira, 14.8 km SW Provincial Road 27 (45°08'13.5"S, 68°10'26.4"W).
Florentino Ameghino Department: LJAMM-CNP 2686–8, Bahía Camarones, Elola Beach (44°50'19.1"S, 65°43'23.1"W); LJAMM-CNP 11421 Garayalde (44°41'22.9"S, 66°37'32.2"W); LJAMM-CNP 11768, National Road 3, 41.4 km N Garayalde (44°24'09"S, 66°18'48"W) ; LJAMM-CNP 9660–9, Garayalde, YPF petrol station (44°41'22.7"S, 66°37'33.3"W); LJAMM-CNP 14309–14337, Cabo Raso, sand dunes near the seashore (44°20'07.3"S, 65°13'46.9"W).
Gastre Department: LJAMM-CNP 6036–8, Mallín area nearby Santana Post, 3 km S Provincial Road 4, 41 km W Gan Gan (42°23'51.2"S, 68°51'22.9"W); LJAMM-CNP 6054–7, Old Post area, 3 km S Provincial Road 4, 37 km W Gan Gan (42°27'43"S, 68°46'56.1"W); LJAMM-CNP 6063, Cerro Navidad area, 3 km S Provincial Road 4, 40 km W Gan Gan (42°24'50.8"S, 68°49'17.5"W); LJAMM-CNP 6067, Aguada Oveja Muerta area, 3 km S Provincial Road 4, 38 km W Gan Gan (42°26'24.6"S, 68°48'20"W).
Languiñeo Department: LJAMM-CNP 4673–4, National Road 25, 5 km W Pampa de Agnia (43°42'24.6"S, 69°43'08.7"W); LJAMM-CNP 6192–4, 6196–6201, National Road 25, 116.8 km N from Pampa de Agnia, near Laguna Aleusco, 2 km S junction Provincial Road 33, Piedra Parada (43°04'50"S, 70°21'20.1"W); LJAMM-CNP 6195, 6202, National Road 25, 5 km W Colan Conhue. Cuesta del Paisano (43°10'39.1"S, 70°01'08.3"W); LJAMM-CNP 8978–80, National Road 25, 44.5 km W Colan Conhue, in front of Laguna Aleusco (43°04'49.9"S, 70°21'20.1"W).
Mártires Department: LJAMM-CNP 10310–19, Provincial Road 29, 34 km E junction Provincial Road 37, road to Garayalde (44°33'09.6"S, 67°26'35.2"W).
Paso de Indios Department: LJAMM-CNP 3720–1, Provincial Road 27, 6.9 km S El Sombrero junction Provincial Road 53 (44°12'21.9"S, 68°14'13.8"W); LJAMM-CNP 3722–4, Provincial Road 27, 14.2 km S El Sombrero and Provincial Road 53 (44°15'33.8"S, 68°15'34.4"W); LJAMM-CNP 3725–8, Provincial Road 27, 42.8 km S El Sombrero, Provincial Road 53 junction (44°28'02.2"S, 68°13'56.8"W); LJAMM-CNP 3729–34, Provincial Road 27, 78.1 km S El Sombrero and Provincial Road 53 (44°35'20.3"S, 67°53'47.2"W); LJAMM-CNP 3774–86, Provincial Road 24, 37 km N junction Provincial Road 23 (44°36'53.6"S, 69°08'33.3"W); LJAMM-CNP 3787–92, Provincial Road 24, 110 km S Paso de Indios (44°31'02.5"S, 69°11'25.9"W); LJAMM-CNP 3800, Provincial Road 27, 68 km S El Sombrero and Provincial Road 53 (44°35'20.4"S, 68°00'57.2"W); LJAMM-CNP 9175–6, Provincial

Road 24, 3 km N Mallín Angosto, 104.7 km N junction Provincial Road 23 (44°31'45.4"S, 69°10'50.3"W); LJAMM-CNP 10270–94, Provincial Road 27, 67.9 km N junction Provincial Road 25, between Meseta Cuadrada and Sierra Cuadrada, 7.9 km S junction Provincial Road 29 (44°40'38.7"S, 67°51'44.2"W).

Río Senguer Department: LJAMM-CNP 2141–4, Provincial Road 20. Sierra de San Bernardo, 19 km W Los Manantiales (45°27'41.1"S, 69°42'52.1"W); LJAMM-CNP 6147–8, Facundo (Provincial Road 43), Senguer River shore (45°09'51.5"S, 70°00'03"W); LJAMM-CNP 11610–5, National Road 40, crossover Senguer River, 49 km NE crossover Mayo River, 2 km SW junction National Road 26 (45°28'10.9"S, 69°49'44.6"W).

Sarmiento Department: LJAMM-CNP 3793–8, Provincial Road 24, 21.2 km N junction Provincial Road 23, N of Laguna Seca (44°44'01.9"S, 69°07'16"W); LJAMM-CNP 3799, Provincial Road 24, 59.2 km N junction Provincial Road 23, in front of Laguna Seca (44°51'57.5"S, 69°13'29.8"W).

Telsen Department: LJAMM-CNP 3203, southern shore of Laguna Sepaucal, towards Sepaucal Agricultural Colony road (Osvaldo Williams Ranch) (42°16'39.0"S, 67°21'17.3"W); LJAMM-CNP 3204, Provincial Road 11, 10.5 km S junction Provincial Road 4 (42°36'37.9"S, 68°05'56.1"W); LJAMM-CNP 3279, Provincial Road 8, 65.7 km S Chubut and Río Negro provinces boundary (42°25'52.9"S, 66°11'13.6"W); LJAMM-CNP 3848–50, 10 km N Gan Gan, entering the first detour from Provincial Road 67 (42°26'44.9"S, 68°18'42.6"W); BYU 47987, Provincial Road 4, 20.4 km E Gan Gan (42°32'08.8"S, 68°01'49.1"W); BYU 47988, Provincial Road 4, 64 km E Gan Gan (42°22'52.1"S, 67°34'48.7"W); BYU 47989–90, 47911, 47992–7, Provincial Road 4, 0.8 km E Gan Gan (42°31'35.8"S, 68°15'29.6"W); BYU 47998, 45.2 km W Telsen (42°22'01.7"S, 67°26'38.8"W); BYU 47999–48000, Provincial Road 4, 2 km E Gan Gan (42°31'44.3"S, 68°01'06.6"W); BYU 48001–3, Provincial Road 4, 65.5 km W Telsen (42°22'03.8"S, 67°39'22"W); BYU 48004, Provincial Road 4, 2 km E Gan Gan (42°31'44.3"S, 68°01'06.6"W); BYU 48005–6, Provincial Road 4, 70.7 km W Telsen (42°22'55.3"S, 67°42'44.8"W); LJAMM-CNP 6041–2, Provincial Road 4, 53 km W Telsen (42°22'06.2"S, 67°30'24.6"W); LJAMM-CNP 6074–6, Provincial Road 4, 18 km E junction Provincial Road 11. 15 km E Gan Gan (42°30'39.9"S, 67°58'41.5"W); LJAMM-CNP 6080, Provincial Road 11, 2.9 km SE Chacay Oeste (42°42'13.9"S, 68°03'34.5"W); LJAMM-CNP 6745, east wayout of Gan Gan, up hills (42°31'04.3"S, 68°16'14"W); LJAMM-CNP 6755, Provincial Road 4, 62.3 km W junction Colonia Sepaucal entrance (Laguna Verde), 28 km E Gan Gan (42°30'37.24"S, 67°58'38.29"W); LJAMM-CNP 6905–6, Provincial Road 67, 16 km N Gan Gan (42°25'27.4"S, 68°17'08.8"W); LJAMM-CNP 7833, Provincial Road 67, 20 km Gan Gan (42°25'10.8"S, 68°14'20.2"W); LJAMM-CNP 8121–2, Laguna de Vaca Road, detour from Provincial Road 4 (42°30'S (67°21'35.6"W); LJAMM-CNP 8133–4, detour from Laguna De Vaca, 16.2 km Provincial Road 4, lagoon shore passing by Elio Calfuquir Post (42°30'S (67°21'35.6"W); LJAMM-CNP 8184, Provincial Road 67, 14 km S Gan Gan, to Laguna Fría (42°38'13.6"S, 68°15'39.7"W); LJAMM-CNP 9191–8, Provincial Road 67, 17.7 km N Gan Gan (2 km detour Cañada Leona) (42°24'18.8"S, 68°15'27.1"W); LJAMM-CNP 13242–6, Road between Cona Niyeu and Telsen, 40.4 km junction Provincial Road 8.

Literature and museum citations:

Kluge (1964): A.M.N.H. Nos. 17001, 46430, for Chubut without specifications; “Provincia” Comodoro Rivadavia: Sarmiento colony, San Bernardo Hills (C.N.H.M. No. 6559).

Biedma Department: Scolaro (1976b): IBA-UNC N° 997, Puerto Pirámide; CNP 09, 17–9, Punta Norte; CNP 23–6, Punta Delgada.

Florentino Ameghino Department: Scolaro (1976a): IBA-UNC N° 1134, CNP 048–9, Galfrascoli Island; CNP 034, CNP 047, Tova Island; CNP 042–4, Isla Tovita.

Río Senguer Department: Specimen examined: BYU 46798, Provincial Road 20, 23 km W Los Manantiales, (45°27'44.6"S, 69°43'26.8"W).

Sarmiento Department: Specimens examined: MCN 1356–9, 24.6 km junction Roads 23 and 24, between Buen Pasto and Paso de Indios.

Scolaro (2005): cited for the entire province.
Conservation status and phytogeographic provinces: not endangered, Provincia Patagónica and Provincia del Monte.

Liolaemidae FROST et al., 2001

Liolaemus bibronii **(**BELL, 1843)
Reference material (Fig. 9):
Cushamen Department**:** LJAMM-CNP 3607–8, Provincial Road 12 and La Cancha Platform (42°47'47.3"S, 70°57'30.2"W); LJAMM-CNP 3671, Provincial Road 15, 1.2 km N junction National Road 40, to Cholila (42°22'24.2"S, 71°07'42.1"W); LJAMM-CNP 3877, Provincial Road 12 and La Cancha Platform (42°47'47.3"S, 70°57'30.2"W); LJAMM-CNP 5681–3, Provincial Road 13, 8 km N El Molle (42°10'24.9"S, 69°32'51.3"W); LJAMM-CNP 5933–5, Provincial Road 13, 8 km N El Molle (42°08'44.7"S, 69°32'37"W); LJAMM-CNP 6277, Provincial Road 4, 23.3 km Cushamen (42°00'15.1"S, 70°40'11.5"W); LJAMM-CNP 8981–5, Provincial Road 12, 9.1 km E La Cancha Platform, to Gualjaina (42°47'44.2"S, 70°51'08.1"W).
Escalante Department: LJAMM-CNP 3878, Provincial Road 25, from Pampa Pelada lowlands of Chico River, 18.4 km SW Provincial Road 27 (45°12'09.4"S, 68°07'21.6"W); LJAMM-CNP 5916, National Road 26, KM 69, 27 km W Pampa del Castillo (45°42'57"S, 68°19'27.4"W); LJAMM-CNP 9909, Provincial Road 25, 20.3 km NW junction Provincial Road 26, La Oración Post (45°20'50.3"S, 68°14'43.8"W).
Florentino Ameghino Department: LJAMM-CNP 11764–7, National Road 3, 41.4 km N Garayalde (44°24'09"S, 66°18'48"W); LJAMM-CNP 2509, Provincial Road 1, 10 km S Dos Pozos (43°58'53"S, 65°25'26"W); LJAMM-CNP 6125, Provincial Road 31, 4 km N junction National Road 3 (44°11'48.9"S, 66°06'55.7"W); LJAMM-CNP 9656–9, Garayalde, YPF petrol station (44°41'22.7"S, 66°37'33.3"W).
Futaleufú Department: LJAMM-CNP 8989, Provincial Road 34, 20.7 km W junction National Road 40 (to Los Rifleros), passing by Lago Cronómetro (43°13'11.9"S, 71°04'46.5"W); LJAMM-CNP 8991–2, National Road 40, 70.6 km N Tecka, km S Nahuel Pan (42°59'24.7"S, 71°05'57.6"W); LJAMM-CNP 8993, Provincial Road 40, 2 km N Esquel Airport entrance (42°53'12.3"S, 71°08'33.8"W); LJAMM-CNP 14357, Laguna La Zeta, Esquel (42°53'42.9"S, 71°20'57.7"W).
Gastre Department: LJAMM-CNP 5940, Provincial Road 50, 23.2 km S Jalalaubat (42°53'36.8"S, 68°40'53.4"W); LJAMM-CNP 5960, Provincial Road 49, 30 km S Gastre (42°31'02.5"S, 69°12'08.5"W); LJAMM-CNP 6033–4, Los Álamos area, 3 km S Provincial Road 4, 40 km W Gan Gan (42°29'39.7"S, 68°43'37.8"W); LJAMM-CNP 6047, Cerro Baritina area, 3 km S Provincial Road 4, 40 km W Gan Gan (42°25'41.8"S, 68°47'49.1"W); LJAMM-CNP 6050–3, Old Post area, 3 km S Provincial Road 4, 40 km W Gan Gan (42°27'43"S, 68°46'56.1"W); LJAMM-CNP 6061, Cerro Navidad area, 3 km S Provincial Road 4, 40 km W Gan Gan (42°24'50.8"S, 68°49'17.5"W); LJAMM-CNP 6064–6, Aguada Oveja Muerta area, 3 km S Provincial Road 4, 40 km W Gan Gan (42°26'24.6"S, 68°48'20"W); LJAMM-CNP 6885, Provincial Road 4, 63 km W Gan Gan (42°23'44.8"S, 68°57'54"W); LJAMM-CNP 9018, Provincial Road 58, 39.6 km NE junction Provincial Road 40, Sierra de la Cicuta (43°07'11"S, 68°37'54.2"W).
Languiñeo Department: LJAMM-CNP 4544–8, National Road 25, 5 km W Pampa de Agnia (43°42'24.6"S, 69°43'08.7"W); LJAMM-CNP 5914, National Road 25, 5 km W Pampa de Agnia (43°44'15.2"S, 69°48'03.8"W); LJAMM-CNP 6181–91, National Road 25, 116.8 km N Pampa de Agnia, nearby Laguna Aleusco entrance, 2 km S Provincial Road 33, Piedra Parada (43°04'50"S, 70°21'20.1"W); LJAMM-CNP 6461, Petrol station, Pampa de Agnia (43°44'55.2"S, 69°39'05.5"W); LJAMM-CNP 8974–7, National Road 25, 44.5 km W Colan Conhue, opposite to Laguna Aleusco (43°04'49.9"S, 70°21'20.1"W); LJAMM-CNP 8986–7, Provincial Road 12, 3 km E Río Gualjaina

bridge, to Piedra Parada (42°39'50.6"S, 70°22'20.1"W); LJAMM-CNP 8988, Provincial Road 33, 7.5 km S Piedra Parada, La Horqueta road junction National Road 25 at Las Golondrinas (42°41'28"S, 70°08'52"W); LJAMM-CNP 9048, 9053–4, Provincial Road 62, 58.9 km E junction National Road 46 (43°35'45.2"S, 70°09'52.9"W).

Mártires Department: LJAMM-CNP 10307–9, Provincial Road 29, 15.5 km E junction Provincial Road 27 to Garayalde (44°34'13.4"S, 67°39'58.4"W); LJAMM-CNP 11066–7, Provincial Road 48 (from Las Plumas to Garayalde), 86.7 km S Las Plumas, first path to Chico River Valley, near La Madreselva Ranch (44°23'54.3"S, 67°01'04.8"W); LJAMM-CNP 3213, National Road 25, 40 km E Las Plumas (43°42'35.4"S, 66°55'04"W); LJAMM-CNP 6120, National Road 25, 35 km E Las Plumas (43°41'34.5"S, 67°02'50.2"W).

Paso de Indios Department: LJAMM-CNP 3735–6, Provincial Road 27, 6.9 km S El Sombrero junction Provincial Road 53 (44°12'21.9"S, 68°14'13.8"W); LJAMM-CNP 3747–51, Provincial Road 27, 14.2 km S El Sombrero and Provincial Road 53 (44°15'33.8"S, 68°15'34.4"W); LJAMM-CNP 3773, Provincial Road 24, 37 km N junction Provincial Road 23 (44°36'53.6"S, 69°08'33.3"W); LJAMM-CNP 8960, Provincial Road 40, 85.7 km SW junction Provincial Road 59 Las Plumas, 10 km E junction Provincial Road 58 (43°28'06.4"S, 68°39'27"W); LJAMM-CNP 8959, 8961–73, Provincial Road 40, 15 km NE junction Provincial Road 12 (43°31'56.8"S, 68°51'06.1"W); LJAMM-CNP 9166–7, Provincial Road 24, 3 km N Mallín Angosto, 104.7 km N junction Provincial Road 23 (44°31'45.4"S, 69°10'50.3"W).

Río Senguer Department: LJAMM-CNP 11609, National Road 40, on the Senguer River junction, 49 km NE Río Mayo junction, 2 km SW junction National Road 26 (45°28'10.9"S, 69°49'44.6"W); LJAMM-CNP 2114, Provincial Road 20, 19 km W Los Manantiales (45°27'41"S, 69°42'52"W); LJAMM-CNP 4550–2, Provincial Road 20, 5 km N Los Tamariscos (44°58'46.8"S, 70°02'53.3"W); LJAMM-CNP 9165, National Road 40, 26 km N Alto Senguer River (44°48'21.9"S, 70°42'24.9"W); LJAMM-CNP 9184–6, National Road 40, 1 km N Pastos Blancos (45°16'13.9"S, 70°31'21.1"W).

Sarmiento Department: LJAMM-CNP 3801, Provincial Road 26, 28.3 km W junction Provincial Road 25, Pampa de los Guanacos, group of rocks opposite to Cerro de los Guanacos (45°22'47.4"S, 68°34'47.2"W); LJAMM-CNP 3805–6, Provincial Road 26, 52.3 km W junction Provincial Road 25, plateau located after Pampa de los Guanacos (45°16'43.9"S, 68°43'03.3"W); LJAMM-CNP 9056–7, Provincial Road 23, 87.8 km SE junction Provincial Road 20, between Los Flamencos and La Blanca Ranchs (44°44'22.3"S, 69°36'29.2"W); LJAMM-CNP 9067–74, 9082–3, Buen Pasto (45°04'08.9"S, 69°27'38.9"W).

Tehuelches Department: LJAMM-CNP 4549, Provincial Road 53, 40 km S junction National Road 25 (43°58'25.2"S, 70°22'05.4"W); LJAMM-CNP 8990 Provincial Road 63, 48 km SW junction Provincial Road 62 (El Molle), 8 km NE José de San Martín (44°00'08.6"S, 70°24'50.8"W); LJAMM-CNP 8994–9, Provincial Road 23, 44.2 km E Provincial Road 20, to Los Flamencos Ranch, 1 km E Los Guindos Ranch (44°35'42.6"S, 69°54'06"W); LJAMM-CNP 9019, Provincial Road 23, to Los Flamencos Ranch, 3.6 km E junction Provincial Road 20 (44°32'18.7"S, 70°21'48.2"W); LJAMM-CNP 9043–7, Provincial Road 23, 44.2 km E Provincial Road 20, to Los Flamencos Ranch, 1 km E Los Guindos Ranch (44°35'42.6"S, 69°54'06"W); LJAMM-CNP 9055, Provincial Road 20, 4 km N Nueva Lubecka Ranch (44°29'07.9"S, 70°25'48.7"W).

Telsen Department: LJAMM-CNP 10989–90, Provincial Road 67, 11.2 km S Chubut and Río Negro boundary (42°04'59.9"S, 68°11'42.3"W); LJAMM-CNP 3222 Provincial Road 4, 35.4 km W Telsen, 4 km E Sepaucal entrance (42°23'46.5"S, 67°20'00.3"W); LJAMM-CNP 3851, 10 km N Gan Gan, taking the first detour from Provincial Road 67 (42°26'44.9"S, 68°18'42.6"W); LJAMM-CNP 5406–8, Provincial Road 4. 0.8 km E Gan Gan (42°31'35.8"S, 68°15'29.6"W); LJAMM-CNP 5474, 9 km W Telsen (42°26'55.3"S, 67°02'42.3"W); LJAMM-CNP 5490–1, Provincial Road 4, 5.4 km W Telsen (42°26'40.4"S, 67°00'06.4"W); LJAMM-CNP 5511–2, Provincial Road 4, 2 km E Gan Gan (42°31'44.3"S, 68°01'06.6"W); LJAMM-CNP 5529, Provincial Road 4, 65.5 km W Telsen

(42°22'03.8"S, 67°39'22"W); LJAMM-CNP 5600–2, Provincial Road 4, 41.6 km W Telsen (42°22'06.9"S, 67°24'07.9"W); LJAMM-CNP 5692, Provincial Road 4, 2 km E Gan Gan (42°31'44.3"S, 68°01'06.6"W); LJAMM-CNP 5911, Quelé Curá (Sierra Colorada), Provincial Road 8 (42°13'38.8"S, 66°21'41.6"W); LJAMM-CNP 5944, Provincial Road 11, 6 km NW Bajada del Diablo (42°52'52.5"S, 67°30'50.9"W); LJAMM-CNP 6058, Provincial Road 4, 11.10 km W Telsen (42°27'00.5"S, 67°03'54.4"W); LJAMM-CNP 6073, Provincial Road 4, 18 km E junction Provincial Road 11, 15 km E Gan Gan (42°30'39.9"S, 67°58'41.5"W); LJAMM-CNP 6081–3, Provincial Road 11, 2.9 km SE Chacay Oeste (42°42'13.9"S, 68°03'34.5"W); LJAMM-CNP 6209–10, Provincial Road 67, 16.5 km N Gan Gan (42°25'06"S, 68°16'39.3"W); LJAMM-CNP 6213, Provincial Road 67, 82 km N Gan Gan (42°04'28.8"S, 68°09'13.4"W); LJAMM-CNP 6485–9, Provincial Road 67, 19.2 km N Gan Gan, Cañada de La Leona (42°06'20.6"S, 68°09'45.9"W); LJAMM-CNP 6735–6, Provincial Road 4, 53.5 km W Telsen, Mallín Grande Ranch (42°21'54.8"S, 67°27'42"W); LJAMM-CNP 6744, east wayout of Gan Gan (42°31'04.3"S, 68°16'14"W); LJAMM-CNP 6937, Provincial Road 4, 25.9 km W Telsen (42°26'20.1"S, 67°14'03.1"W); LJAMM-CNP 6940–1, detour road from Laguna de Vaca, 3.5 km S junction Provincial Road 4 (42°26'52.6"S, 67°18'51.6"W); LJAMM-CNP 8117–20, 8123–4, Laguna de Vaca road, detour Provincial Road 4 (42°30'00"S, 67°21'35.6"W); LJAMM-CNP 8137, Provincial Road 8, 55.2 km junction Provincial Road 5 (42°11'51.8"S, 66°22'47.4"W); LJAMM-CNP 8189, Provincial Road 67, 10 km W junction Provincial Road 11, 58 km E junction Provincial Road at Laguna Fría (36 km S Gan Gan) (42°52'16"S, 68°03'51.2"W); LJAMM-CNP 13629–33, Provincial Road 4, 10.2 km W Telsen (42°27'02.4"S, 67°03'43.6"W).
Literature and museum citations:
Daciuk & Miranda (1980): cited without department. Cei (1986): present on the Atlantic shores of Chubut and colonized the islands Tova, Tovita and Galfrascoli (IBA-UNC 1107 Tova Island; CNP 050 Tovita Island; CNP 051 Galfrascoli Island; Scolaro, (1976a). Scolaro (2005) cited for almost the entire province.
Conservation status and phytogeographic provinces: not endangered, Provincia Patagónica and Provincia del Monte.

Liolaemus boulengeri KOSLOWSKY, 1896
Reference material (Fig. 9):
Cushamen Department: LJAMM-CNP 3609–16, 3676–7, Provincial Road 12, La Cancha platform (42°47'47.3"S, 70°57'30.2"W); LJAMM-CNP 3886, Fofo Cahuel (Netchovitch´s Ranch) (42°19'54.7"S, 70°33'14.6"W); LJAMM-CNP 7074–5, Provincial Road 4, 8.4 km SE Chico River area, Bajada del Platero (42°02'04"S, 70°18'31,5"W); LJAMM-CNP 7083–6, Provincial Road 4, 23.3 km Cushamen (42°00'15.1"S, 70°40'11.5"W).
Escalante Department: LJAMM-CNP 10204–6, Provincial Road 26, 21.1 km NW junction Provincial Road 37, 29.1 km NW junction National Road 26, 3.9 km SW Rancho Grande Ranch (45°33'29.6"S, 68°11'08.9"W); LJAMM-CNP 10215–25, Provincial Road 26, junction Provincial Road 26, 36 km NW junction Provincial Road 37, Pampa Vaca (45°29'42.7"S, 68°18'30.3"W); LJAMM-CNP 10244–64, Pampa Pelada, 8.1 km junction Provincial Road 25, to Sierra Chaira, 14.8 km SW Provincial Road 27 (45°08'13.5"S, 68°10'26.4"W); LJAMM-CNP 10639, Provincial Road 26, junction Provincial Road 26, 36 km NW junction Provincial Road 37, Pampa Vaca (45°29'42.7"S, 68°18'30.3"W); LJAMM-CNP 3767, Provincial Road 27, 46.5 km S junction Provincial Road 29 (a Garayalde) (44°56'07.6"S, 68°01'37"W); LJAMM-CNP 4593, National Road 3, 44 km N Comodoro Rivadavia (45°33'10.4"S, 67°37'28.87"W); LJAMM-CNP 9200, National Road 3, 70.2 km SW Garayalde (45°07'39.7"S, 67°08'49.6"W); LJAMM-CNP 9676, Provincial Road 37, 2.5 km W junction National Road 3 (45°37'43.4"S, 67°41'03.6"W).
Florentino Ameghino Department: LJAMM-CNP 9648, Garayalde, YPF petrol station (44°41'22.7"S, 66°37'33.3"W).

Gastre Department: LJAMM-CNP 10942, Provincial Road 4, 59.3 km W Gan Gan (42°23'08.7"S, 69°03'38.9"W).
Languiñeo Department: LJAMM-CNP 12168, National Road 25, 23.1 km E El Molle, 8 km W Pampa de Agnia (43°41'30.5"S, 69°46'04.1"W); LJAMM-CNP 2845, National Road 25, 5 km W Pampa de Agnia (43°44'15.2"S, 69°48'03.8"W); LJAMM-CNP 3476, National Road 40, 2 km S Tecka, junction Provincial Road 62 (43°30'26.8"S, 70°48'06.8"W); LJAMM-CNP 3887, Piedra Parada (Campo de Creton) (42°42'59.4"S, 70°03'52.6"W); LJAMM-CNP 4728–30, National Road 25, 5 km W Pampa de Agnia (43°42'24.6"S, 69°43'08.7"W); LJAMM-CNP 8847–50, 8853–56, Provincial Road 12, 3 km E bridge over Gualjaina River, to Piedra Parada (42°39'50.6"S, 70°22'20.1"W); LJAMM-CNP 8872, 9049–52, Provincial Road 62, 58.9 km E junction National Road 40 (43°35'45.2"S, 70°09'52.9"W).
Mártires Department: LJAMM-CNP 11059–61, Provincial Road 48 from Las Plumas to Garayalde, 25.5 km S Las Plumas (43°56'09.4"S, 67°18'08.7"W); LJAMM-CNP 11062–65, Provincial Road 48 from Las Plumas to Garayalde, 78 km S Las Plumas, 17.1 km S La Madreselva Ranch (44°20'59.9"S, 67°05'44.1"W); LJAMM-CNP 13665–6, Provincial Road 11, 5 km E local road (connected to Provincial Road 4, at 20 km from Telsen) (43°00'42.4"S, 67°15'22.6"W).
Paso de Indios Department: LJAMM-CNP 11131–2, Provincial Road 27, 35.8 km NW junction Provincial Road 29 in Bosque Petrificado (44°30'17.1"S, 68°11'36.9"W); LJAMM-CNP 3718, Provincial Road 24, 37 km N junction Provincial Road 23 (44°36'53.6"S, 69°08'33.3"W); LJAMM-CNP 3752–59, 3869–70 Provincial Road 27, 14.2 km S El Sombrero and Provincial Road 53 (44°15'33.8"S, 68°15'34.4"W); LJAMM-CNP 9115, Provincial Road 24, 3 km N Mallín Angosto, 104.7 km N junction Provincial Road 23 (44°31'45.4"S, 69°10'50.3"W); LJAMM-CNP 9136, Provincial Road 24, 153.5 km N junction Provincial Road 23, Cañadón Grande (44°13'26.8"S, 69°19'49.3"W).
Sarmiento Department: LJAMM-CNP 3802, Provincial Road 26, 28.3 km W junction Provincial Road 25, Pampa de los Guanacos, group of rocks opposite to Cerro de los Guanacos (45°22'47.4"S, 68°34'47.2"W); LJAMM-CNP 3810–13, Provincial Road 26, 52.3 km W junction Provincial Road 25, plateau located after Pampa de los Guanacos (45°16'43.9"S, 68°43'03.3"W); LJAMM-CNP 3864, Provincial Road 26, 6.9 km W junction Provincial Road 25 (nearby Cerro Guacho) (45°28'19.4"S, 68°22'35.7"W).
Tehuelches Department: LJAMM-CNP 4689–95, Provincial Road 53, 40 km S junction National Road 25 (43°58'25.2"S, 70°22'05.4"W); LJAMM-CNP 8888, Provincial Road 19, 9 km W junction National Road 40, to Pico River (44°01'53.2"S, 70°51'10.3"W); LJAMM-CNP 8889, Provincial Road 63, 48 km SW junction Provincial Road 62 (El Molle), 8 km NE José de San Martín (44°00'08.6"S, 70°24'50.8"W).
Telsen Department: LJAMM-CNP 3429–30, Provincial Road 67, 30.3 km N Gan Gan (Sitio Aguadas) (42°23'36.1"S, 68°12'22.1"W); LJAMM-CNP 5925, Provincial Road 67, 80.8 km Gan Gan (42°04'29.8"S, 68°09'38.7"W); LJAMM-CNP 6922–3, Provincial Road 67, 10 km N Gan Gan (42°26'33.99"S, 68°19'21.34"W); LJAMM-CNP 6939, Provincial Road 4, 92 km W Telsen approximately 40 km E Gan Gan (42°28'26.1"S, 67°51'35.4"W).
Literature and museum citations:
Koslowsky (1898): MLP S 843, S 1047–1048, 95–96, 2172, 2177–2180, 2182–2183. S 799 (Series type), 842 (Lectotype) for "Neuquén and Chubut territories". Cited for Chubut without details by Cei (1986).
Biedma Department: Scolaro (1976b): IBA-UNC N° 937, Salinas Grandes; CNP 04–5, La Adela Ranch. Daciuk & Miranda (1980): CENAI 1765 (JD-Z 1708), Nuevo Gulf, Punta Dorado; CENAI 1766 (JD-Z 1642), San Jorge Gulf, in front of Quintano Island.
Gaiman Department: Daciuk & Miranda (1980): CENAI 279, between 28 de Julio and Ameghino dam. Scolaro (2005): cited for the entire province. Abdala (2005): refers to the area from the type locality for this species restricted to Esquel, Trevelín, Tecka and Gobernador Costa.

Futaleufú Department: Abdala (2005): FML 15733–738, 6 ej. 6 km E junction between National Road 40 and Provincial Road 12, on Provincial Road 12.
Languiñeo Department: Cited by Abdala (2005) FML 15712–713, 2 ej. 75 km S Esquel, 200 m from Arroyo Canquel, on National Road 40; FML 15714–718, 5 ej. 6 km S Tecka on National Road 40; FML 15722–724, 3 ej. 1 km from junction between National Road 40 and Provincial Road 17, on Provincial Road 17, to Corcovado; FML 15740–746, 7 ej. 100 m junction National Road 40 and Provincial Road 17, on Provincial Road 17, to Corcovado; FML 15711, 1 ej. Pocitos de Quichaura; FML 15747–749, 3 ej. 11 km E Pocitos de Quichaura; FML 15707–710, 4 ej. 4 km al SE junction between National Road 40 and Provincial Road 62 on Provincial Road 62.
Tehuelches Department: Abdala (2005): FML 15725–732, 8 ej. 6 km N of Gobernador Costa, on National Road 40; FML 15719–721, 3 ej. 72 km N of Esquel, on National Road 40.
Cushamen Department: Abdala (2005): MACN 26194, El Maitén, Cushamen Department; MACN 23347–52, Arroyo Perdido; MACN 15308, El Maitén.
Florentino Ameghino Department: Specimens examined: MCN 782, km marker 1594 on National Road 3, 1 km N Uzcudun (44°15.917'S, 66°09.074'W).
Río Senguer Department: Specimens examined: MCN 1339–40, Los Tamariscos, Road 20.
Conservation status and phytogeographic provinces: not endangered, Provincia Patagónica and Provincia del Monte.

Liolaemus camarones ABDALA, DÍAZ-GÓMEZ & JUAREZ-HEREDIA, 2012
Reference material (Fig. 10):
Florentino Ameghino Department: LJAMM-CNP 11736–9, 11744–6, Provincial Road 1, 31 km S Camarones, 1 km E La Isabel Ranch entrance, shore of a temporal stream with sandy soils (44°54'55.1"S, 65°59'19"W); BYU 48154, MLP.S 2469, LJAMM-CNP 2464, BYU 48155, LJAMM-CNP 2500, FML 13052, LJAMM-CNP 2502–4, Camarones Bay, Elola Beach (44°50'19"S, 65°43'23"W).
Literature and museum citations:
Florentino Ameghino Department: Abdala et al. (2012b): FML 23301–08, Beach 3 km south of Camarones, Bahia Camarones; FML 13965, 18 km south of Camarones; FML 24140–4, beach 1 km south of Camarones, road to Elola beach (44°48'33.00"S, 65°44'8.90"W).
Conservation status and phytogeographic provinces: not endangered, Provincia Patagónica.

Liolaemus canqueli CEI, 1975
Reference material (Fig. 10):
Gastre Department: LJAMM-CNP 2927, Provincial Road 12, nearby Cerro Gorro Frigio, 53 km S Paso del Sapo (43°08'05.1"S, 69°17'43.5"W).
Languiñeo Department: LJAMM-CNP 11083–4, on the road to 6 Hermanos Ranch, between junction National Road 25 in Pampa de Agnia and Colan Conhue, 25 km N Pampa de Agnia (43°36'03.4"S, 69°50'39.2"W); LJAMM-CNP 11085–6, Old National Road 25, Epulef Village entrance, 12 km S Colan Conhue (43°19'41.8"S, 69°52'24.8"W); LJAMM-CNP 12166–7, National Road 25, 23.1 km E El Molle, 8 km W Pampa de Agnia (43°41'30.5"S, 69°46'04.1"W); BYU 47283–4, LJAMM-CNP 2900, MLP.S 2470 Pampa de Agnia (43°45'18.6"S, 69°38'27.7"W); LJAMM-CNP 2902–4, Pampa de Agnia (43°45'18.6"S, 69°38'27.7"W); LJAMM-CNP 4665–72, National Road 25, 5 km W Pampa de Agnia (43°42'24.6"S, 69°43'08.7"W); LJAMM-CNP 12918–25, National Road 25, Los Pocitos de Quichaura entrance, 28 km NW El Molle (43°38'07.5"S, 70°06'31"W); LJAMM-CNP 8878–87, Provincial Road 62, 58.9 km E junction National Road 40 (43°35'45.2"S, 70°09'52.9"W).
Mártires Department: LJAMM-CNP 11049–58, Provincial Road 48 (Las Plumas to Garayalde), 25.5 km S Las Plumas (43°56'09.4"S, 67°18'08.7"W); LJAMM-CNP 2148, MLP.S 2467–8, BYU 48115, LJAMM-CNP 2227, LJAMM-CNP 2256, FML 13053, Valle de los Mártires, National Road 25, km

249, 19 km E junction Provincial Road 27 (43°49'41"S, 67°45'21"W); LJAMM-CNP 4613–4, Las Plumas (43°43'34"S, 67°17'27.5"W); LJAMM-CNP 3211–2, National Road 25, 7.5 km W Las Plumas (43°43'18.7"S, 67°22'30.6"W).
Paso de Indios Department: LJAMM-CNP 11081, Provincial Road 27, 35.8 km NW junction Provincial Road 29 (Bosques Petrificados) (44'30'17.1"S, 68°11'36.9"W); LJAMM-CNP 12357–61, Provincial Road 53, 6.1 km SE Paso de Indios (43°53'29.8"S, 68°58'28.8"W); LJAMM-CNP 12362–4, 12366–70 Provincial Road 53, 9 km SE Paso de Indios (43°54'23.4"S, 68°57'00.6"W); LJAMM-CNP 12365, 12371, 12374–5, 12377–8, Provincial Road 53, 13.9 km SE Paso de Indios (43°54'43.1"S, 68°55'13.7"W); LJAMM-CNP 12372–3, 12376, Provincial Road 53, 13.2 km SE Paso de Indios (43°54'50.6"S, 68°54'57"W); LJAMM-CNP 12379, Provincial Road 53, 17.5 km SE Paso de Indios (43°55'30.6"S, 68°52'17"W); LJAMM-CNP 2149, Provincial Road 53, 14.1 km SE Paso de Indios (43°54'46.3"S, 68°54'25.6"W); LJAMM-CNP 2199, FML 13047, LJAMM-CNP 2230–2, Provincial Road 53, 20.7 km SE Paso de Indios (43°56'20"S, 68°50'14"W); LJAMM-CNP 2270–1, 2286, Provincial Road 53, 8 km SE Paso de Indios (43°53'52"S, 68°57'27"W); BYU 48129, BYU 47280–1, LJAMM-CNP 2930, MLP.S 2472, LJAMM-CNP 2932, 4889, Provincial Road 12, 6 km S Cerro Cóndor (43°27'38.9"S, 69°08'38.2"W); LJAMM-CNP 3470–5, Provincial Road 53, 48.3 km NW El Sombrero, 37 km SE Paso de Indios (43°59'46"S, 68°40'22"W); LJAMM-CNP 3742, Provincial Road 27, 6.9 km S El Sombrero junction Provincial Road 53 (44°12'21.9"S, 68°14'13.8"W); LJAMM-CNP 4602–8, National Road 25, 15 km W Los Altares (43°50'50.4"S, 68°32'50.3"W); LJAMM-CNP 4725–7, National Road 25, 8 km W Paso de Indios (43°50'46.4"S, 69°09'32.1"W); LJAMM-CNP 9102–9, Provincial Road 24, 24.4 km N Laguna de La Bombilla (43°59'40.3"S, 69°08'36.5"W); LJAMM-CNP 11618–20, Provincial Road 27, 2.4 km S junction National Road 25 (43°52'26.2"S, 67°58'03.9"W); LJAMM-CNP 3453–8, Provincial Road 27, 13.8 km S National Road 25, El Sombrero road (43°56'26"S, 68°01'50.7"W); LJAMM-CNP 8811–17, Provincial Road 12, 6.2 km N Cerro Cóndor nearby Cañadón de Los Loros (43°23'13.9"S, 69°10'14"W).
Literature and museum citations:
Cei (1973a): Callejas at the north of Meseta Canquel. Cei (1986): cited for an area limited by Chubut River on the north, to Pampa de Agnia, Meseta de Sotomayor and the salty deserts of Payahilé on the west. Scolaro (2005): Meseta Canquel and surroundings.
Paso de Indios Department: Abdala (2007): FML 2874, 2915, 2786, Provincial Road 53, 20 km SE Paso de los Indios; FML 13966–967, El Sombrero Plateau; MCN 1288–1295, Hills in front of El Sombrero, El Sombrero, Canquel Plateau; REE 812–818, W El Sombrero Plateau, 1.2 km N junction Provincial Road 53 and Provincial Road 90, km 1778; MCN 14.1, El Sombrero. Specimens examined: FBC 0008–21, 46 km SE Paso de Indios, Provincial Road 535 (44°03.771'S, 68°35.657'W); BYU 46769, Junction Provincial Roads 27 and 29, 106 km W Garayalde (44°36'15.1"S, 67°48'37.4"W).
Conservation status and phytogeographic provinces: not endangered, Provincia Patagónica and Provincia del Monte.

Liolaemus chehuachekenk AVILA, MORANDO & SITES, 2008
Reference material (Fig. 10):
Cushamen Department: MLP.S 2535–6, FML 15105, BYU 48202–3, FML 15106, Provincial Road 13, 8 km N El Molle (42°10'24.9"S, 69°32'51.3"W); LJAMM-CNP 5926–32, Provincial Road 13, 8 km N El Molle (42°08'44.7"S, 69°32'37"W); LJAMM-CNP 6742–3, Provincial Road 4, 47.6 km W Gan Gan (42°24'58.6"S, 68°47'35.8"W).
Gastre Department: LJAMM-CNP 12327, 12356, 12383–99, 12409–12, 12959, Provincial Road 58, 13.5 km N El Escorial (42°58'34.5"S, 68°34'35.6"W); LJAMM-CNP 5936–9, Provincial Road 50, 10 km N El Escorial (43°00'18.2"S, 68°34'14.1"W); LJAMM-CNP 5961–5, Provincial Road 49, 30 km S Gastre (42°31'02.5"S, 69°12'08.5"W); LJAMM-CNP 8825–30, Provincial Road 58, 23 km SW junction Provincial Road 59, sandy soils in El Colorado ditch (43°13'31.6"S, 68°38'20"W); LJAMM-CNP

8832–3, Provincial Road 58, 39.6 km NE junction Provincial Road 40 (43°07'11"S, 68°37'54.2"W).
Languiñeo Department: LJAMM-CNP 8851, Provincial Road 12, 3 km E bridge over Gualjaina River, to Piedra Parada (42°39'50.6"S, 70°22'20.1"W).
Paso de Indios Department: LJAMM-CNP 12344–7, 12349–55, 12380–2, 12402–8, 13348, Provincial Road 40, 60 km W El Caramelo Ranch, 10 km E junction Provincial Road 58 (43°27'27.4"S, 68°40'39.8"W).
Telsen Department: LJAMM-CNP 3412–4, Provincial Road 67, 7.5 km N Gan Gan (42°27'02.7"S, 68°19'49.1"W); LJAMM-CNP 3418–20, Provincial Road 67, 12.5 km N Gan Gan (42°25'30.9"S, 68°17'59.9"W); LJAMM-CNP 3852–55, 3872, 10 km N Gan Gan, taking the first detour from Provincial Road 67 (42°26'44.9"S, 68°18'42.6"W); LJAMM-CNP 5425, Provincial Road 4, 0.8 km E Gan Gan (42°31'35.8"S, 68°15'29.6"W); LJAMM-CNP 5465, Provincial Road 4, 74 km E Gan Gan (42°21'48.2"S, 67°29'29.1"W); LJAMM-CNP 6738–40, Provincial Road 4, 53.5 km W Telsen, Mallín Grande Ranch (42°21'54.8"S, 67°27'42"W); LJAMM-CNP 6750, Provincial Road 67, 7 km N Gan Gan (42°27'58.4"S, 68°19'39.4"W); LJAMM-CNP 6753–4, Provincial Road 4, 62.3 km W junction Colonia Sepaucal entrance (Laguna Verde), 28 km E Gan Gan (42°30'37.2"S, 67°58'38.2"W); LJAMM-CNP 6758–9, Provincial Road 67, 11.2 km N Gan Gan (42°27'42.7"S, 68°18'42.6"W); LJAMM-CNP 6886, Provincial Road 67, 16 km N Gan Gan (42°25'04.9"S, 68°16'47,5"W); LJAMM-CNP 6927, Provincial Road 67, 10 km N Gan Gan (42°26'49.7"S, 68°19'44.4"W); LJAMM-CNP 6935–6, Provincial Road 4, 25.9 km W Telsen (42°26'20.1"S, 67°14'03.1"W); LJAMM-CNP 7852–69, to Laguna de Vaca from Provincial Road 4, 4 km S detour (42°33'03.2"S, 68°06'56"W); LJAMM-CNP 8136, Laguna de Vaca detour, 16.2 km Provincial Road 4, lagoon surroundings, passing by Elio Calfuquir Post (42°30'00"S, 67°21'35.6"W); BYU 49355–72, Laguna de Vaca road, detour from Provincial Road 4 (42°35'13.9"S, 68°06'31.9"W); LJAMM-CNP 8171–6, 8182, Laguna de Vaca road, from south of Provincial Road 4 (42°35'13.9"S, 68°06'31.9"W); LJAMM-CNP 13252, Road between Cona Niyeu and Telsen city, 58.6 km from Provincial Road 8 (42°19'38.2"S, 66°55'45.2"W); LJAMM-CNP 13634–6, LJAMM-CNP 13681–5, LJAMM-CNP 13699–712, LJAMM-CNP 13855, 13857, Provincial Road 4, 10.2 km W Telsen (42°27'02.4"S, 67°03'43.6"W); LJAMM-CNP 13655–8, Local Road, 43 km S junction Provincial Road 4 (connected to Provincial Road 4, 20 km Telsen) (42°42'36.7"S, 67°01'22"W).
Conservation status and phytogeographic provinces: not endangered, Provincia Patagónica and Provincia del Monte.

Liolaemus darwinii (BELL, 1843)
Reference material (Fig. 10):
Biedma Department: LJAMM-CNP 13140–8, 13150–3, Cerro Avanzado (42°50'40.3"S, 64°52'22.3"W); LJAMM-CNP 2466–9, 2619, 2621, Provincial Road 42, 11 km NE Puerto Madryn, El Doradillo Beach (42°39'25"S, 64°59'32"W); FML13064, Puerto Madryn (42°47'36,4"S, 64°58'15,3"W); MLP.S 2485, Puerto Madryn (42°47'36,4"S, 64°58'15,3"W); LJAMM-CNP 4585–8, Puerto Madryn, El Doradillo Beach (42°39'46.88"S, 64°59'33.5"W); LJAMM-CNP 6112–5, Provincial Road 4, 8 km W junction National Road 3 (42°44'19.2"S, 65°12'50.2"W); LJAMM-CNP 6920–1, Puerto Madryn (42°44'53.1"S, 65°03'37.9"W); BYU 48817, Puerto Madryn (42°44'53.1"S, 65°03'37.9"W); LJAMM-CNP 7523, Península Valdés, El Centro (42°12'13"S, 63°58'33"W); LJAMM-CNP 13611–3, Provincial Road 60, 11.2 km E junction National Road 3 (42°03'00.9"S, 65°09'54.6"W); LJAMM-CNP 13617–8, Road to El Bajo del Gualicho, 28 km junction National Road 3 (42°21'54.7"S, 65°30'26.2"W); LJAMM-CNP 13619–20, Road to El Bajo del Gualicho, 13 km junction National Road 3 (42°21'35.5"S, 65°20'08.1"W); LJAMM-CNP 13645–8, Provincial Road 1, 21.6 km N junction Provincial Road 2 (42°25'52.8"S, 65°04'18.7"W).
Gaiman Department: LJAMM-CNP 6916–7, Provincial Road 8, 28 km NW Trelew (43°02'45.4"S, 65°27'57.2"W); LJAMM-CNP 13277–9, Provincial Road 40, 20.6 km junction Provincial Road 25 (43°12'37.2"S, 65°59'40.1"W); LJAMM-CNP 13284, Unpaved road 3 km from its access on Provincial

Road 40, 31.6 km junction Provincial Road 25 (43°08'36.8"S, 66°07'18.4"W); LJAMM-CNP 13284, Local Road, 3km N junction Provincial Road 40 (43°08'36.8"S, 66°07'18.4"W).
Mártires Department: LJAMM-CNP 13664, Provincial Road 11, 5 km E from local road (connected to Provincial Road 4, 20 km Telsen) (43°00'42.4"S, 67°15'22.6"W).
Rawson Department: LJAMM-CNP 5500–3, 10 km NW Trelew (43°07'57.6"S, 65°22'47.3"W).
Telsen Department: LJAMM-CNP 5472–3, 24 km E Trelew (42°31'48.2"S, 66°42'19.8"W); LJAMM-CNP 5945–6, 6133–5, Provincial Road 61, 40.3 km junction Provincial Road 11, between Ranquilhuao and San Manuel Ranchs (42°44'48.8"S, 66°59'54.8"W); LJAMM-CNP 13211–2, Provincial Road 8, 73.1 km NW junction Provincial Road 4 (42°18'31.7"S, 66°16'00.6"W); LJAMM-CNP 13218, Provincial Road 8, 82.6 km junction Provincial Road 4 (42°14'54.5"S, 66°20'00.9'W); LJAMM-CNP 13637–44, Local Road, 25 km S junction Provincial Road 4, 20 km E Telsen (42°37'57.3"S, 66°56'41.5"W); LJAMM-CNP 13659–60, Local Road, 43 km S junction Provincial Road 4, 20 km E Telsen (42°42'36.7"S, 67°01'22"W).
Literature and museum citations:
Biedma Department: Scolaro (1976b): IBA-UNC N° 1160, Fracasso Ranch; CNP 02, Ameghino isthmus. Cited by Daciuk & Miranda (1980): CENAI 731–2, road to Punta Lobos; CENAI 1762 (JD-Z 1350), Puerto Pirámide; CENAI 1767, Punta Norte, Península Valdés. Cei (1986): cited reaching south Península Valdés. Scolaro (2005): Biedma and Rawson Departments. Abdala (2007): MACN 31766–67, Península Valdés. Specimens examined: FBC 0062–5, Playa Paraná, S Puerto Madryn (42°47.921'S, 64°56.487'W).
Telsen Department: Abdala (2007): MACN 35912–13, Chata Sierra. Specimens examined: MCN 1327–8, 64 km N junction between Roads 8 and 4 (Sierra Colorada).
Conservation status and phytogeographic provinces: not endangered, Provincia Patagónica and Provincia del Monte.

Liolaemus elongatus KOSLOWSKY, 1896
Reference material (Fig. 10):
Cushamen Department: LJAMM-CNP 3452, 3711–4, Provincial Road 15, 1.2 km W junction National Road 40, to Cholila (42°22'24.2"S, 71°07'42.1"W); LJAMM-CNP 3631–2, La Hoya (ski resort), 13 km NE Esquel (42°50'03.7"S, 71°15'29.1"W); LJAMM-CNP 8197, Cerro Ferarroti (42°31'21.3"S, 70°02'23.4"W); LJAMM-CNP 8852, Provincial Road 12, 9.1 km E La Cancha Platform, to Gualjaina (42°47'44.2"S, 70°51'08.1"W).
Futaleufú Department: LJAMM-CNP 2128–9, 2156–7, National Road 40, km 1530, 17 km S Esquel, 5 km junction National Road 40 and National Road 259 (42°58'06.5"S, 71°09'55"W); LJAMM-CNP 2164, 2168–9, National Road 40, 18 km N Tecka, km 1589 (43°20'42"S, 70°52'40"W); LJAMM-CNP 2262, National Road 40, km 1530, 17 km S Esquel, 5 km S junction National Road 40 and National Road 259 (42°58'06.5"S, 71°09'55"W); LJAMM-CNP 3574–80, Provincial Road 17, 8.8 km SE Corcovado (43°30'20.7"S, 71°23'44.3"W); LJAMM-CNP 8871, Provincial Road 34, 20.7 km W junction National Road 40 (road of los Rifleros), passing by Lago Cronómetro (43°13'11.9"S, 71°04'46.5"W); LJAMM-CNP 8890, Provincial Road 40, 2 km N Esquel Airport entrance (42°53'12.3"S, 71°08'33.7"W).
Languiñeo Department: LJAMM-CNP 12926–7, National Road 25, Los Pocitos de Quichaura entrance, 28 km NW El Molle (43°38'07.5"S, 70°06'31"W); LJAMM-CNP 13129–30, Provincial Road 13, 5 km N Laguna Blanca, to Paso del Sapo (42°54'05.7"S, 69°53'32.3"W); LJAMM-CNP 3898, Provincial Road 62, 15.2 km N El Molle, junction Provincial Road 63 (Sierra de Languiñeo) (43°37'34.7"S, 70°06'45.6"W); LJAMM-CNP 6177–80, National Road 25, 5 km W Colan Conhue, Cuesta del Paisano (43°10'39.1"S, 70°01'08.3"W); LJAMM-CNP 8873–6, Provincial Road 62, 58.9 km E junction National Road 40 (43°35'45.2"S, 70°09'52.9"W).
Paso de Indios Department: LJAMM-CNP 3715–9, 3831, Provincial Road 24, 110 km S Paso de Indios

(44°31'02.5"S, 69°11'25.9"W); LJAMM-CNP 9100, Provincial Road 24, 146.7 km N junction Provincial Road 23, 2 km E Cañadón Grande Ranch entrance (44°15'17.1"S, 69°23'47.3"W); LJAMM-CNP 9110–4, Provincial Road 24, 3 km N Mallín Angosto, 104.7 km N junction Provincial Road 23 (44°31'45.4"S, 69°10'50.3"W).
Río Senguer Department: LJAMM-CNP 3046–7, FML 13070, Provincial Road 20, 23 km W Los Manantiales (45°42'40.5"S, 70°15'49.2"W); LJAMM-CNP 4873, Provincial Road 20, 23 km W Los Manantiales (45°27'44.6"S, 69°43'26.8"W).
Sarmiento Department: LJAMM-CNP 9059–66, Provincial Road 23, 87.8 km SE junction Provincial Road 20, between Los Flamencos and La Blanca Ranchs (44°44'22.3"S, 69°36'29.2"W); LJAMM-CNP 9075–8, Buen Pasto (45°04'08.9"S, 69°27'38.9"W); LJAMM-CNP 9087–92 Provincial Road 23, Sierra del Castillo, 37.5 km NW junction Provincial Road 26, and 27.5 km NW junction Provincial Road 24 Paso de Indios, 5 km NW Matasiete (45°07'10"S, 69°20'01.6"W).
Tehuelches Department: LJAMM-CNP 3049, National Road 40, 22 km S Gobernador Costa (44°12'08.2"S, 70°26'44.6"W); FML 13071, National Road 40, 22 km S Gobernador Costa (44°12'08.2"S, 70°26'44.6"W); LJAMM-CNP 3905–10, National Road 40, 45.0 km SW junction Provincial Road 20, passing by Arroyo El Puma (44°31'06.6"S, 70°36'48.7"W); LJAMM-CNP 3911–8, National Road 40, 19.6 km SW junction Provincial Road 20, before La Providencia Ranch entrance (44°23'20.6"S, 70°37'17.9"W); LJAMM-CNP 3934, Provincial Road 19, 2.7 km junction National Road 40, 16 km W Gobernador Costa (44°00'23.9"S, 70°48'13.7"W); LJAMM-CNP 4681–3, Provincial Road 53, 40 km S junction National Road 25 (43°58'25.2"S, 70°22'05.4"W); LJAMM-CNP 6145–6, Near Gobernador Costa (44°14'26.5"S, 70°25'27.5"W).
Telsen Department: LJAMM-CNP 10974–6, Provincial Road 67, 11.2 km S Chubut and Río Negro provinces boundary (42°04'34.4"S, 68°09'43.1"W); LJAMM-CNP 7514, Provincial Road 67, Talagapa road, 53.1 km N Gan Gan (42°13'50.8"S, 68°14'23.8"W).
Literature and museum citations:
Futaleufú Department: Cei (1974): cited as *Liolaemus elongatus elongatus*, IBA-UNC 598, 1, Nahuel Pan, Esquel; 602, 1, 18 km N Tecka; 855, 1–2, 2 km N Costa; 609, 1–2, 5 km SE Tamariscos. Cited by Scolaro (2005) along a strip that runs along the eastern side of the province.
Tehuelches Department: Specimens examined: BYU 46731–2 National Road 40, 22 km S Gobernador Costa (44°12'08.2"S, 70°26'44.6"W).
Languiñeo Department: Specimens examined: MCN 780, Pocitos de Quichuara, 43.2 km W Pampa de Agnia, 60.2 km SE junction National Road 40 and Provincial Road 62 on Provincial Road 62 (43°37.882' S, 70°06.647' W).
Sarmiento Department: MCN 1322, Sierra de Castillo 27 km junction Roads 24 and 25, to Buen Pasto; 1325–6, Las Pulgas (opposite Gruta de la Virgen); 1343–4, 1389–92, 27 km junction Roads 24 and 25, to Buen Pasto, Sierra de Castillo.
Conservation status and phytogeographic provinces: not endangered, Provincia Patagónica and Provincia Subantártica.

Liolaemus fitzingerii (DUMÉRIL & BIBRON, 1837)
Reference material (Fig. 10):
Escalante Department: LJAMM-CNP 10214 Provincial Road 26, 36 km NW junction Provincial Road 37, Pampa Vaca (45°29'42.7"S, 68°18'30.3"W); LJAMM-CNP 10226–30 Provincial Road 25, 20.3 km NW junction Provincial Road 26, La Oración Post (45°20'50.3"S, 68°14'43.8"W); LJAMM-CNP 10243 Pampa Pelada, 8.1 km junction Provincial Road 25, to Sierra Chaira, 14.8 km SW Provincial Road 27 (45°08'13.5"S, 68°10'26.4"W); LJAMM-CNP 11074–7 Provincial Road 27 (Nollman bridge road, over Chico River), 3.4 km NW junction National Road 3 (45°30'21.8"S, 67°37'27.2"W); LJAMM-CNP 11747–50 Bustamante Bay, 1 km S Alguera Soriano Sociedad Anónima, to Puerto Visser (45°07'10.5"S, 66°33'33"W); LJAMM-CNP 11751–55 Provincial Road 1, 49.8 km S Bustamante Bay,

between La Perseverancia and La Aurora Ranchs (45°17'20.1"S, 67°01'33"W); LJAMM-CNP 11756–7 Provincial Road 1, nearby La Máquina Ranch, passing by Puerto Visser Ranch, 74.6 km SW Bustamante Bay (45°20'01.2"S, 67°04'32.6"W); BYU 47288, LJAMM-CNP 2879 National Road 26, km 69, 27 km W Pampa del Castillo (45°42'57"S, 68°19'27.4"W); LJAMM-CNP 3768–71, 3880–1 Provincial Road 27, 46.5 km S junction Provincial Road 29 (to Garayalde) (44°56'07.6"S, 68°01'37"W); LJAMM-CNP 3844–6, 3867 Provincial Road 27, 65.4 km S junction Provincial Road 29 (to Garayalde) (45°05'14.1"S, 68°01'31.2"W); LJAMM-CNP 3875–6 Provincial Road 25, Pampa Pelada lowlands of Chico River, 18.4 km SW Provincial Road 27 (45°12'09.4"S, 68°07'21.6"W); LJAMM-CNP 4888 National Road 26, km 29, 27 km W Pampa del Castillo (45°42'57"S, 68°19'27.4"W).

Florentino Ameghino Department: LJAMM-CNP 11758–63, National Road 3, 41.4 km N Garayalde (44°24'09"S, 66°18'48"W); LJAMM-CNP 2204, Provincial Road 32, 4 km before Provincial Road 2 junction (44°01'55"S, 65°30'37"W); FML 13048, Provincial Road 32, 4 km before Provincial Road 2 junction (44°01'55"S, 65°30'37"W); LJAMM-CNP 2220, 2527–30, 1 km S Dos Pozos, Provincial Road 1 (43°54'37"S, 65°24'10"W); LJAMM-CNP 2221–2, 2.5 km N Dos Pozos, Provincial Road 1 (43°53'15"S, 65°25'51"W); MLP.S 2460, 20 km S junction of Provincial Road 32 and Provincial Road 1 (44°10'27"S, 65°25'22"W); BYU 48113–4, LJAMM-CNP 2427–8, LJAMM-CNP 2505–8, MLP.S 2528, LJAMM-CNP 2658, Provincial Road 1, 10 km S Dos Pozos (43°58'53"S, 65°25'26"W); LJAMM-CNP 2491–5, Cabo Raso (44°19'23"S, 65°15'46"W); LJAMM-CNP 6121–3, Provincial Road 31, 36 km S Florentino Ameghino dam (43°51'58.3"S, 66°09'53.7"W); LJAMM-CNP 6124, Provincial Road 31, 4 km N junction National Road 3 (44°11'48.9"S, 66°06'55.7"W); LJAMM-CNP 8152, Camino Cabo Dos Bahías, 19.2 km S Camarones, Caleta Pedro (44°53'14.2"S, 65°38'06.7"W); LJAMM-CNP 9644–7, Garayalde, YPF petrol station (44°41'22.7"S, 66°37'33.3"W); LJAMM-CNP 14304–8, unpaved road towards the shore, 8 km N Cabo Raso, next to the beach (44°16'12.9"S, 65°16'32.6"W); LJAMM-CNP 14338–40, Cabo Raso, sand dunes near shore (44°20'07.3"S, 65°13'46.9"W).

Paso de Indios Department: LJAMM-CNP 9117–9, Provincial Road 24, 83.9 km N junction Provincial Road 23 (44°41'00.7"S, 69°07'10.2"W).

Río Senguer Department: MLP.S 2462, FML 13050, Provincial Road 20, 19 km W Los Manantiales (45°27'41"S, 69°42'52"W); LJAMM-CNP 2888, Provincial Road 20, 3 km N La Laurita, 30 km S Nueva Lubecka (44°44'37.3"S, 70°12'48.2"W); LJAMM-CNP 2889, National Road 40, 2 km S Mayo River (45°42'40.5"S, 70°15'49.2"W); LJAMM-CNP 3919–24, Provincial Road 51, 25.2 km S Ricardo Rojas, passing by Arroyo Chalia, surroundings of Laguna Quilchamal (45°43'35"S, 70°58'45.8"W); LJAMM-CNP 3925–8, Provincial Road 26, 68 km SW junction Provincial Road 38, in opposite direction to Mayo River Valley (45°37'26.3"S, 70°50'05.3"W); LJAMM-CNP 3935–7, Provincial Road 26, 4.4 km SW junction Provincial Road 38, Mayo River Valley (45°36'23.6"S, 70°49'31.2"W); LJAMM-CNP 4576, Provincial Road 20, 5 km N Los Tamariscos (44°58'46.8"S, 70°02'53.3"W); LJAMM-CNP 4890, Provincial Road 20, 30 km S Nueva Lubecka, 3 km N La Laurita (44°44'37.3"S, 70°12'48.2"W).

Sarmiento Department: MLP.S 2459, LJAMM-CNP 2215, FML 13049, 24 km SE Sarmiento, Bosque Petrificado road (45°47'45"S, 69°04'12"W); LJAMM-CNP 3865, Provincial Road 26, 6.9 km W junction Provincial Road 25 (nearby Cerro Guacho) (45°28'19.4"S, 68°22'35.7"W); LJAMM-CNP 9079–81 Buen Pasto (45°04'08.9"S, 69°27'38.9"W); LJAMM-CNP 9139–44, Provincial Road 23, 31.8 km SE Buen Pasto, 7.5 km NW junction Provincial Road 24 (45°12'47.9"S, 69°11'01.4"W); LJAMM-CNP 9145–50, Laguna Seca, north shore, Provincial Road 24, 53.1 km N junction Provincial Road 23 (44°50'15.3"S, 69°13'21.9"W).

Tehuelches Department: LJAMM-CNP 2921–2, 2925, National Road 40, 7.9 km SW junction Provincial Road 20 (44°21'57.7"S, 70°29'31.2"W); BYU 47308, BYU 47291, National Road 40, 7.9 km SW junction Provincial Road 20 (44°21'57.7"S, 70°29'31.2"W); LJAMM-CNP 9007–11, Provincial

Road 23, 44.2 km E Provincial Road 20, to Los Flamencos Ranch, 1 km E Los Guindos Ranch (44°35'42.6"S, 69°54'06"W); LJAMM-CNP 9017, Provincial Road 23, to Los Flamencos Ranch, 30 km E junction Provincial Road 20, at the Cerro Ferraroti base (44°31'45.1"S, 70°01'41.2"W); LJAMM-CNP 9020, Provincial Road 23, to Los Flamencos Ranch, 3.6 km E junction Provincial Road 20 (44°32'18.7"S, 70°21'48.2"W).
Literature and museum citations:
Cei (1973a): for southern Chubut and part of Santa Cruz, between 44° and 55° S. Scolaro (1976a): for Tova (IBA-UNC N° 1097, CNP 054–7), Tovita (IBA-UNC N° 1135, CNP 059) and Galfrascoli (IBA-UNC N° 1136) islands along the coasts of Chubut province.
Biedma Department: Daciuk and Miranda (1980): cited this species for Península Valdés.
Scolaro (2005): cited for the southeastern area and a vertical N-S narrow strip in Chubut.
Florentino Ameghino Department: Abdala (2007): FML 2130, Isla Galfrasconi, opposite Meló Pier, 35 km SW Camarones.
Rawson Department: Abdala (2007): FML 2870, 2783, 2705, Provincial Road 32, junction Provincial Road 1.
Florentino Ameghino Department: Abdala (2007): FML 13968–69, km 1954 1 km N Uzcudún, National Road 3; CSA 372, 60 km to the South of Garayalde, National Road 3; REE 883–88, 3.1 km N Uzcudún. Specimens examined: FBC 0022–32, Between Provincial Roads 1 and 32, S from Dos Pozos, (43°55.308'S, 65°24.416'W).
Escalante Department: Specimens examined: MACN 3335–7, Comodoro Rivadavia.
Conservation status and phytogeographic provinces: not endangered, Provincia Patagónica and Provincia del Monte.

Liolaemus gracilis (BELL, 1843)
Reference material (Fig. 10):
Biedma Department: LJAMM-CNP 13012, Fracasso Beach, Península Valdés (42°25'45.6"S, 64°07'51.4"W); LJAMM-CNP 2620, Provincial Road 42, 11 km NE Puerto Madryn. El Doradillo Beach (42°39'25"S, 64°59'32"W); LJAMM-CNP 3325–9, Salinas Chicas, Península Valdés (42°38'43.8"S, 63°50'30"W); LJAMM-CNP 3330–42, San Pablo Ranch (42°36'54"S, 64°10'26"W); LJAMM-CNP 3345, El Centro Ranch, Península Valdés (42°12'13"S, 63°58'53"W); LJAMM-CNP 3698–9, Puerto Madryn (42°46'37.6"S, 65°01'21.7"W); LJAMM-CNP 4926, Salinas Chicas (42°39'17.3"S, 63°51'47.1"W); LJAMM-CNP 5980–2, Puerto Madryn (CENPAT) (42°47'06.3"S, 65°00'30.8"W); LJAMM-CNP 6310, 6318–9, 6348, 6359, Salinas Chicas, Península Valdés (42°38'45.6"S, 63°51'46.8"W); LJAMM-CNP 6324–34, 6342–5, 6357, 6371–6, 6386–7, San Pablo Ranch, Península Valdés (42°37'53.7"S, 64°08'23.2"W); LJAMM-CNP 6360–1, 7587–9, El Trébol Ranch, Península Valdés (42°43'20.4"S, 63°53'44.5"W); LJAMM-CNP 6942, Puerto Madryn, seaside promenade (42°46'41.8"S, 65°01'16"W); LJAMM-CNP 7522, Península Valdés, El Centro (42°12'13"S, 63°58'33"W); LJAMM-CNP 7543–4, 7564, 7577–9, El Progreso Ranch, Península Valdés (42°37'52.6"S, 64°08'31.7"W); LJAMM-CNP 7566–7, Los Médanos Ranch (42°42'04.2"S, 64°06'06.9"W); LJAMM-CNP 7596–7, Salinas Chicas (42°38'59.8"S, 63°51'53.8"W).
Gaiman Department: LJAMM-CNP 13276, Provincial Road 40, 20.6 km junction Provincial Road 25 (43°12'37.2"S, 65°59'40.1"W).
Telsen Department: LJAMM-CNP 5486, Provincial Road 4, 3.5 km W Telsen (42°26'27.8"S, 66°58'50.6"W); LJAMM-CNP 5947, Provincial Road 61, 40.3 km junction Provincial Road 11, between Ranquilhuao and San Manuel Ranchs (42°44'48.8"S, 66°59'54.8"W); LJAMM-CNP 13219–20, Provincial Road 8, 82.6 km junction Provincial Road 4 (42°14'54.5"S, 66°20'00.9"W; Minoli & Avila 2010).
Literature and museum citations:
Biedma Department: Scolaro (1976b): for Península Valdés, IBA-UNC N°961, Punta Delgada; CNP

N°09–10, La Cantábrica Ranch; CNP N° 76 Punta Morro Nuevo. Cei (1986): for the ecotonal area Monte-Patagonian Steppe, from the northeastern part of the province towards the Río Chubut, with a possible southern distribution. Scolaro (2005): cited for a north-south narrow strip, along coastline departments.

Rawson Department: Specimens examined: MCN 1345, 40 km N from Trelew.

Conservation status and phytogeographic provinces: not endangered, Provincia Patagónica and Provincia del Monte.

Liolaemus kingii (BELL, 1843)

Reference material (Fig. 11):

Cushamen Department: LJAMM-CNP 3678–9, Provincial Road 12 and La Cancha Platform (42°47'47.3"S, 70°57'30.2"W).

Escalante Department: LJAMM-CNP 10189–10200, Holdich Station (45°58'00.1"S, 68°11'58.8"W); LJAMM-CNP 10231–42, 10640, Pampa Pelada, 8.1 km junction Provincial Road 25, to Sierra Chaira. 14.8 km SW Provincial Road 27 (45°08'13.5"S, 68°10'26.4"W); LJAMM-CNP 11418–20, Holdich Station (45°57'58.4"S, 68°12'00.4"W); LJAMM-CNP 13017–8, Provincial Road 37, 22.8km SW junction National Road 3 (45°41'10.6"S, 67°53'49.9"W); LJAMM-CNP 13027–9, Holdich Station (45°58'00.7"S, 68°11'58.9"W); LJAMM-CNP 4589–92, National Road 3, 44 km N Comodoro Rivadavia (45°33'10.4"S, 67°37'28.8"W); LJAMM-CNP 9201–3, National Road 3, 70.2 km SW Garayalde (45°07'39.7"S, 67°08'49.6"W); LJAMM-CNP 9205–9, 9632–3, Holdich Station (45°57'59.9"S, 68°11'58.5"W); LJAMM-CNP 9671–5, 9778, Provincial Road 37, 2.5 km W junction National Road 3 (45°37'43.4"S, 67°41'03.6"W).

Futaleufú Department: LJAMM-CNP 8877, National Road 40, 70.6 km N Tecka, km S Nahuel Pan (42°59'24.7"S, 71°05'57.6"W); LJAMM-CNP 8891–3, Provincial Road 40, 2 km N Esquel Airport entrance (42°53'12.3"S, 71°08'33.7"W).

Languiñeo Department: LJAMM-CNP 11098, Provincial Road 13, 20.3 km N Colan Conhue, Paso del Sapo road (43°04'16.8"S, 69°53'47.6"W); LJAMM-CNP 13083–8, 6 Hermanos Ranch, 10 km N-NE junction National Road 25, on the way of Provincial Road 62, Pocitos de Quichaura (43°26'23.9"S, 70°00'12.7"W); LJAMM-CNP 13106–13, Provincial Road 13, 18.8 km N Colan Conhue (43°04'52.5"S, 69°53'58.1"W); LJAMM-CNP 13114–7, Laguna Blanca, Provincial Road 13 between Colan Conhue and Paso del Sapo, 34.2 km N Colan Conhue (42°57'14.4"S, 69°55'47.7"W); LJAMM-CNP 3680, 3701–2, Provincial Road 17, 52.5 km SE Corcovado (43°37'42.5"S, 70°59'49.8"W); LJAMM-CNP 6501–2, Provincial Road 44, 35.9 km S Corcovado (43°45'56.4"S, 71°23'52.4"W); LJAMM-CNP 9183, National Road 40, 16.1 km S Tecka (43°37'47.7"S, 70°50'27.2"W).

Paso de Indios Department: LJAMM-CNP 8896–9, 9012–16, 9029–37, 9094–99, Provincial Road 23, 65.1 km E junction Provincial Road 20, Los Flamencos Ranch road (44°35'50.7"S, 69°41'26.1"W); LJAMM-CNP 9101, Provincial Road 24, 5.8 km N Laguna La Bombilla (44°06'22.8"S, 69°16'13.6"W); LJAMM-CNP 9130–5, Provincial Road 24, 153.5 km N junction Provincial Road 23, passing by Cañadón Grande (44°13'26.8"S, 69°19'49.3"W); LJAMM-CNP 9189–90, Provincial Road 23, 77.6 km E junction Provincial Road 20, 1 km SE Los Flamencos Ranch (44°39'58.2"S, 69°36'22.5"W).

Río Senguer Department: LJAMM-CNP 13062–6, National Road 40, junction National Road 260, Lago Blanco road (45°56'45.9"S, 70°24'12.5"W); LJAMM-CNP 13070–1, National Road 40, 2 km S Mayo River (45°42'57.4"S, 70°15'53"W); LJAMM-CNP 2121–2, 2145–6, 2211–3, 2217–8, 2308–9, Provincial Road 20, 4 km N junction Provincial Road 22 (45°25'54"S, 69°50'25"W); LJAMM-CNP 3938–45, 3960–3, LJAMM-CNP 3995–6, Provincial Road 51, 2.8 km S Dr. Ricardo Rojas (45°35'53.2"S, 71°02'54"W); LJAMM-CNP 3964–71, LJAMM-CNP 3993–4, Provincial Road 38, 28.8 km S Arroyo Verde Ranch, downhill to Cañadón Tacho (45°20'01.5"S, 70°52'05.8"W); LJAMM-CNP

3977, Provincial Road 51, 49.5 km S Doctor Ricardo Rojas, junction Provincial Road 55 (45°52'15"S, 71°05'57"W); LJAMM-CNP 3978–80, Provincial Road 38, 14.6 km SW Alto Río Senguer, passing Ciervo Rojo entrance, 1 km N Verde Stream Ranch (45°04'50.6"S, 70°59'02.6"W); LJAMM-CNP 4698, National Road 40, 3.5 S Mayo River (45°43'27.2"S, 70°16'16.8"W); LJAMM-CNP 6500, Provincial Road 38, 56.1 km S Arroyo Verde Ranch, passing by Cañadón Alto Ranch, 6.4 km junction Provincial Road 26 (45°32'57.5"S, 70°49'50.4"W); LJAMM-CNP 6505–7, Provincial Road 51, 25.2 km S Dr. Ricardo Rojas, passing Chalia Stream, Laguna Quilchamal (45°43'35"S, 70°58'45.8"W); LJAMM-CNP 8894, 9151–8, National Road 40, 26 km N Alto Río Senguer (44°48'21.9"S, 70°42'24.9"W); LJAMM-CNP 9181, National Road 26, 22.3 km NW junction Provincial Road 18 (45°57'25.5"S, 69°52'08.9"W).
Sarmiento Department: LJAMM-CNP 3807–9, 3891–2, Provincial Road 26, 52.3 km W junction Provincial Road 25, plateau after the ascent from Pampas of the Guanacos (45°16'43.9"S, 68°43'03.3"W); LJAMM-CNP 9042, 9120–29, Provincial Road 23, Sierra del Castillo, 37.5 km NW junction Provincial Road 26 and 27.5 km NW junction Provincial Road 24, Paso de Indios, 5 km NW Matasiete (45°07'10"S, 69°20'01.6"W).
Tehuelches Department: LJAMM-CNP 4684–8, Provincial Road 53, 40 km S junction National Road 25 (43°58'25.2"S, 70°22'05.4"W).
Literature and museum citations:
Escalante Department: Cited by Scolaro & Cei (1997).
Scolaro (2005): cited for southern Chubut and a vertical strip over the central and western areas of the province.
Sarmiento Department: Lobo (2005): MCZ 11837, 39–40, Patagonia; MCN 1324 Las Pulgas (hills opposite of Gruta de la Virgen).
Río Senguer Department: Specimen examined: BYU 46777 National Road 40, 2 km S Río Mayo, (45°42'40.5"S, 70°15'49.2"W).
Paso de Indios Department: Specimens examined: MCN 1354–5, 1393, 60 S from Paso de los Indios, Road 24.
Languiñeo Department: Specimens examined: MCN 1557–64, 4.0 km SE junction National Road 40 and Provincial Road 62 on Provincial Road 62 (43°30.925'S; 70°45.584'W).
Conservation status and phytogeographic provinces: not endangered, Provincia Patagónica and Provincia Subantártica.

Liolaemus kriegi Müller & Hellmich, 1939
Reference material (Fig. 11):
Literature and museum citations:
Cushamen Department: Cruz et al. (1999): CRILaR PT 4848–50, Provincial Road 13, 25 km S Moligüe (42°04'S; 69°31'W).
Conservation status and phytogeographic provinces: not endangered, Provincia Patagónica.

Liolaemus lineomaculatus BOULENGER, 1885
Reference material (Fig. 11):
Futaleufú Department: LJAMM-CNP 3664, Provincial Road 17, 26.6 km SE Corcovado (43°30'56.4"S, 71°13'04.9"W); LJAMM-CNP 3685, 3687, Provincial Road 17, 36.4 km SE Corcovado (43°33'43.8"S, 71°10'01.3"W).
Languiñeo Department: LJAMM-CNP 9182, National Road 40, 16.1 km S Tecka (43°37'47.7"S, 70°50'27.2"W).
Tehuelches Department: LJAMM-CNP 9093, Provincial Road 19, 9 km W junction National Road 40, to Pico River (44°01'53.2"S, 70°51'10.3"W).
Literature and museum citations:

Río Senguer Department: Williams (1997): MLP.S. 1669–70, for the southern shore of the west end of Lago Fontana (44°51'S, 71°40'W).
Conservation status and phytogeographic provinces: not endangered, Provincia Patagónica and Provincia Subantártica.

Liolaemus morandae BREITMAN, PARRA, PÉREZ & SITES, 2011
Reference material (Fig. 11):
Escalante Department: LJAMM-CNP 9677–80, Provincial Road 37, 2.5 km W junction National Road 3 (45°37'43.4"S, 67°41'03.6"W); LJAMM-CNP 13020–21, Provincial Road 37, 22.8 km SW junction National Road 3 (45°41'10.6"S, 67°53'49.9"W); LJAMM-CNP 10201–2, Holdich Station (45°58'00.1"S, 68°11'58.8"W).
Río Senguer Department: LJAMM-CNP 3998, Provincial Road 38, 28.8 km S Arroyo Verde Ranch, downhill to Cañadón Tacho (45°20'01.5"S, 70°52'05.8"W).
Literature and museum citations
Escalante Department: Breitman et al. (2011): MLP.S 2626, Provincial Road 37, 22.8km SW junction National Road 3 (45°41'10.6"S, 67°53'49.9"W).
Conservation status and phytogeographic provinces: not endangered, Provincia Patagónica.

Liolaemus melanops BURMEISTER, 1888
Reference material (Fig. 11):
Biedma Department: LJAMM-CNP 11044–7, Puerto Pirámide (42°34'31.5"S, 64°16'07.6"W); LJAMM-CNP 13009–11, Fracasso Beach, Península Valdés (42°25'45.6"S, 64°07'51.4"W); LJAMM-CNP 13149, Cerro Avanzado (42°49'57.8"S, 64°52'58.5"W); LJAMM-CNP 2415–6, Provincial Road 42, 7 km N Punta Flecha (42°36'44.4"S, 64°54'13.8"W); LJAMM-CNP 2617–8, Provincial Road 42, 11 km NE Puerto Madryn. El Doradillo Beach (42°39'25"S, 64°59'32"W); LJAMM-CNP 2942–45, 3041–2, Puerto Madryn (42°47'36.4"S, 64°58'15.3"W); LJAMM-CNP 3421, Cerro Avanzado, 10 km S Puerto Madryn (42°49'57.4"S, 64°52'58.5"W); LJAMM-CNP 3451, Cerro Avanzado (42°49'57.4"S, 64°52'58.5"W); LJAMM-CNP 4513–4, Puerto Madryn (42°44'46.6"S, 65°02'20.3"W); LJAMM-CNP 6111, Provincial Road 4, 8 km W junction National Road 3 (42°44'21.3"S, 65°12'32.8"W); LJAMM-CNP 7519–21, Península Valdés, El Centro (42°12'13"S, 63°58'33"W); LJAMM-CNP 8151 Punta Ameghino Beach, 15 km N de El Doradillo Beach (42°35'38.8"S, 64°48'51.6"W).
Gaiman Department: LJAMM-CNP 5952–8, Provincial Road 11, 55 km N junction National Road 25, 5 km S Provincial Road 40 (43°18'53"S, 67°00'59.7"W); LJAMM-CNP 6918–9, Provincial Road 8, 28 km NW Trelew (43°02'45.4"S, 65°27'57.2"W); LJAMM-CNP 13274, Provincial Road 40, 2.5 km junction Provincial Road 25 (43°17'21"S, 65°49'22.6"W).
Mártires Department: LJAMM-CNP 11797, National Road 25, km 143, 64 km E Las Plumas (43°38'12"S, 66°38'39"W); LJAMM-CNP 12180, 12181–6, Provincial Road 40, 2 km W junction Provincial Road 59 (43°28'59.7"S, 67°44'37.1"W); LJAMM-CNP 12996–13000, National Road 25, 42.3 km E Las Plumas (43°42'19.4"S, 66°52'51.9"W); LJAMM-CNP 3214, National Road 25, 40 km E Las Plumas (43°42'35.4"S, 66°55'04"W); LJAMM-CNP 6119, National Road 25, 35 km E Las Plumas (43°41'34.5"S, 67°02'50.2"W); LJAMM-CNP 8779–84, Provincial Road 11, 29.1 km NW junction National Road 25, 1 km NW Mina Chubut (43°28'11.3"S, 66°50'55.8"W); LJAMM-CNP 8785–9, Provincial Road 40, 40.2 km W junction Provincial Road 11 between El Alba and El Sauce Ranchs (43°23'09.6"S, 67°31'46.2"W); LJAMM-CNP 8790, Provincial Road 40, 3 km SW junction Provincial Road 59 Las Plumas (43°29'15.8"S, 67°45'15.8"W).
Paso de Indios Department: LJAMM-CNP 8791, Provincial Road 40, 9.1 km SW junction Provincial Road 59 Las Plumas (43°30'13.2"S, 67°49'30.3"W); LJAMM-CNP 8792–8810, Provincial Road 40, 34.8 km E junction Provincial Road 58, passing La Rosa Ranch (43°27'38.4"S, 68°24'11.4"W);

LJAMM-CNP 8837–46, Provincial Road 40, 15 km NE junction Provincial Road 12 (43°31'56.8"S, 68°51'06.1"W).
Rawson Department: LJAMM-CNP 11770–1, Bajo de los Huesos, 15.7 km junction Provincial Road 1 (43°11'49.8"S, 64°52'00"W).
Telsen Department: LJAMM-CNP 11153–63 Provincial Road 8, 112 km NW junction Provincial Road 4, 1 km S Aguada Malaspina Ranch (42°02'22"S, 66°34'16.5"W); LJAMM-CNP 11189–95, road between Cona Niyeu and Telsen, 41.8 km S junction Provincial Road 8 (in Río Negro) (42°12'05.2"S, 66°52'07.9"W); LJAMM-CNP 11196–98, road between Cona Niyeu and Telsen, 53.3 km S junction Provincial Road 8 (in Río Negro) (42°16'41.9"S, 66°54'32.3"W); LJAMM-CNP 2934, BYU 48152, MLP.S 2473–4, BYU 48153, LJAMM-CNP 2939–41, Provincial Road 8, in Quelé Curá or Sierra Colorada (42°13'38.8"S, 66°21'41.6"W); LJAMM-CNP 5941–3, Provincial Road 11, 6 km NW Bajada del Diablo (42°52'52.5"S, 67°30'50.9"W); LJAMM-CNP 5948–51, Provincial Road 61, 40.3 km junction Provincial Road 11, between Ranquilhuao and San Manuel Ranchs (42°44'48.8"S, 66°59'54.8"W); LJAMM-CNP 6734, Provincial Road 4, 100 km W Puerto Madryn, 76 km E Telsen (42°42'15.7"S, 66°10'46.2"W); LJAMM-CNP 6895–6, Provincial Road 4, 56 km W junction Provincial Road 8 (42°37'45.7"S, 66°22'28.5"W); LJAMM-CNP 6929–33, Provincial Road 4, 63.2 km W Sierra Chata, 30 km E Telsen (42°31'27.8"S, 66°38'03.5"W); LJAMM-CNP 8109–16, Provincial Road 8, 49 km S junction Provincial Road 5 (42°08'59.2"S, 66°24'55.1"W); LJAMM-CNP 13213–5, Provincial Road 8, 73.1 km NW junction Provincial Road 4 (42°18'31.7"S, 66°16'00.6"W); LJAMM-CNP 13216–7, LJAMM-CNP 13550–68, Provincial Road 8, 82.6 km junction Provincial Road 4 (42°14'54.5"S, 66°20'00.9'W); LJAMM-CNP 13686–98, Road between Cona Niyeu and Telsen, 40.4 km junction Provincial Road 8 (42°12'10.2"S, 66°51'22.6"W).
Literature and museum citations:
Cited for south of Chubut River by Cei (1973a; b).
Biedma Department: Scolaro (1976b): cited this species for Península Valdés, Punta Norte, Punta Delgada and La Cantábrica Ranch. Specimen examined: BYU 46770, Puerto Madryn (42°47'36.4"S, 64°58'15.3"W); BYU 46741, Provincial Road 8, 18 km N junction Provincial Road 4 (42°37'22.2"S, 65°54'49.9"W).
Daciuk & Miranda (1980): CENAI 854 (JD-Z 1734), Sierra Cuadrada, Canquel.
Telsen Department: Specimen examined: MCN 1297–1301, 1304–6, Sierra Colorada, 70 km junction Roads 8 and 4.
Mártires Department: Specimen examined: MCN 1312–4, 2 km E Las Plumas.
Rawson Department: Specimen examined: MCN 1332–3, 40 km N Trelew, Road 3.
Conservation status and phytogeographic provinces: not endangered, Provincia Patagónica and Provincia del Monte.

Liolaemus petrophilus DONOSO-BARROS & CEI, 1971
Reference material (Fig. 11):
Cushamen Department: LJAMM-CNP 3218–20, Country road 27.1 km W Provincial Road 13, passing by Laguna Del Toro, 13 km Provincial Road 66 (42°21'52.7"S, 69°56'24.8"W); LJAMM-CNP 7076–80, Provincial Road 4, 8.4 km SE Chico River place, Bajada del Platero (42°02'04"S, 70°18'31.5"W).
Gastre Department: LJAMM-CNP 6043–4, area Viejo Post, 3 km S Provincial Road 4, 40 km W Gan Gan (42°27'43"S, 68°46'56.1"W).
Languiñeo Department: FML 13063, BYU 47097, Provincial Road 12, 8 km S Paso del Sapo (42°48'11.5"S, 69°33'58.1"W); LJAMM-CNP 3885, Piedra Parada (Creton Field) (42°41'27.6"S, 70°02'12.9"W).
Mártires Department: FML 13046, LJAMM-CNP 2126, 2264, Mártires Valley, National Road 25, km 249, 19 km E junction Provincial Road 27 (43°49'41"S, 67°45'21"W); LJAMM-CNP 4615–6, Las

Plumas (43°43'34"S, 67°17'27.5"W).
Paso de Indios Department: LJAMM-CNP 3058, FML 13074, Provincial Road 12, 6 km N Cerro Cóndor, 72 km N Paso de Indios (43°27'38.9"S, 69°08'38.2"W); LJAMM-CNP 3465–9, Provincial Road 27 and Provincial Road 53, hill opposite Cerro El Sombrero (44°09'09.9"S, 68°14'46"W); LJAMM-CNP 3762–6, Provincial Road 27, 78.1 km S El Sombrero and Provincial Road 53 (44°35'20.3"S, 67°53'47.2"W); LJAMM-CNP 3772 Provincial Road 24, 37 km N junction Provincial Road 23, high plateau (44°36'53.6"S, 69°08'33.3"W); LJAMM-CNP 3884, Provincial Road 12, 50 km S Cerro Frigio (Campo de Pichiñan) (43°33'19.1"S, 69°04'04.8"W); MLPS 2604, Provincial Road 12, 6 km N Cerro Cóndor, 72 km N Paso de Indios (43°23'18.4"S, 69°10'13.1"W).
Sarmiento Department: LJAMM-CNP 3803–4, Provincial Road 26, 28.3 km W junction Provincial Road 25, Pampa de los Guanacos, rock groups in front of Cerro de los Guanacos (45°22'47.4"S, 68°34'47.2"W).
Telsen Department: FML 13079, LJAMM-CNP 4874, Provincial Road 8 at Quelé Curá (Sierra Colorada) (42°13'38.8"S, 66°21'41.6"W); LJAMM-CNP 3215–7, Provincial Road 4, 93.7 km W Telsen, 25.3 km E Provincial Road 11 (42°29'16.8"S, 67°53'49.6"W); LJAMM-CNP 3411, Provincial Road 67, 17.7 km N Gan Gan (2 km detour Cañada Leona) (42°24'18.8"S, 68°15'27.1"W); LJAMM-CNP 3814–22, Provincial Road 67, 31.4 km N Gan Gan (Sitio El Lloradero) (42°22'57.9"S, 68°10'45.4"W); LJAMM-CNP 5437–9, Provincial Road 4, 20.4 km E Gan Gan (42°32'08.8"S, 68°01'49.1"W); LJAMM-CNP 5481–5, 5583, Provincial Road 4, 56.9 km W Telsen (42°22'36.6"S, 67°34'00"W); LJAMM-CNP 5504, Provincial Road 4, 60.4 km W Telsen (42°22'35.4"S, 67°35'42.9"W); LJAMM-CNP 5513–9, Provincial Road 4, 2 km E Gan Gan (42°31'44.3"S, 68°01'06.6"W); LJAMM-CNP 5594–5, Provincial Road 4, 41.6 km W Telsen (42°22'06.9"S, 67°24'07.9"W); LJAMM-CNP 6039–40, Provincial Road 4, 53 km W Telsen (42°22'06.2"S, 67°30'24.6"W); LJAMM-CNP 6072, Provincial Road 4, 18 km E junction Provincial Road 11, 15 km E Gan Gan (42°30'39.9"S, 67°58'41.5"W); LJAMM-CNP 6077–9, Provincial Road 11, 2.9 km SE Chacay Oeste (42°42'13.9"S, 68°03'34.5"W); LJAMM-CNP 6110, Provincial Road 4, Laguna Verde, 20 km E Gan Gan, 101.4 km W Telsen (42°30'37.5"S, 67°58'38.3"W); LJAMM-CNP 6204–6, Provincial Road 67, 16.5 km N Gan Gan (42°25'06"S, 68°16'39.3"W); LJAMM-CNP 6493, Provincial Road 67, 20.7 km N Gan Gan (42°25'01.4"S, 68°12'45.6"W); LJAMM-CNP 6737, Provincial Road 4, 53.5 km W Telsen, Mallín Grande Ranch (42°21'54.8"S, 67°27'42"W); LJAMM-CNP 6756, Provincial Road 67, 17.6 km N Gan Gan (42°25'27.8"S, 68°17'07.7"W); LJAMM-CNP 6898–6902, Provincial Road 67, 16 km N Gan Gan (42°25'27.4"S, 68°17'08.8"W); LJAMM-CNP 6953–9, 7102, Provincial Road 4, 23 km E Gan Gan (42°32'06.1"S, 68°01'29.9"W); LJAMM-CNP 7835, 7839–51, Provincial Road 67, 20 km N Gan Gan (42°25'10.8"S, 68°14'20.2"W); LJAMM-CNP 8135, detour Laguna De Vaca, 16.2 km Provincial Road 4, at the lagoon shore passing by Elio Calfuquir Post (42°30'00"S, 67°21'35.6"W); LJAMM-CNP 8185, Provincial Road 67, 22.6 km S Gan Gan, Laguna Fría road (42°41'45.8"S, 68°13'57.6"W); LJAMM-CNP 13253–6, Road between Cona Niyeu and Telsen city, 58.6 km from Provincial Road 8 (42°19'38.2"S, 66°55'45.2"W).
Literature and museum citations:
Scolaro (1993): CNP-CH 431, for Villa Dique Ameghino (43°50'S, 67°20'W).
Mártires Department: Schulte II et al. (2004): for National Road 25, Las Chapas.
Paso de Indios Department: Specimens examined: PT 4841–3, Provincial Road 13, N de Paso de Indios (42°26.066'S, 69°41.147'W); PT 4838–40, 59 km SE Paso de Indios, Provincial Road 53 (44°07.513'S, 68°32.212'W); MCN 1346–7, opposite Cerro Sombrero.
Conservation status and phytogeographic provinces: not endangered, Provincia Patagónica and Provincia del Monte.

Liolaemus pictus argentinus (DUMÉRIL & BIBRON, 1837)
Reference material (Fig. 12):

Languiñeo Department: LJAMM-CNP 6515–6, Provincial Road 44, 24.6 km S Corcovado (43°42'08.1"S, 71°24'37.6"W; Avila et al. 2006a).
Futaleufú Department: LJAMM-CNP 14343, W from walking path over Arrayanes river, Los Alerces National Park (42°43'33.6"S, 71°44'32.9"W).
Literature and museum citations:
Cited as *Liolaemus pictus argentinus* for the northwest corner of Chubut by Cei (1986) and Scolaro (2005).
Conservation status and phytogeographic provinces: not endangered, Provincia Subantártica.

Liolaemus rothi KOSLOWSKY, 1898
Reference material (Fig. 12):
Cushamen Department: LJAMM-CNP 6834, Provincial Road 13, 17.7 km S Chubut and Río Negro boundary, Sierras de Calcatapul (42°04'25.2"S, 69°30'54.2"W); LJAMM-CNP 6981, Provincial Road 4, 56.3 km SE Chico River area, 1 km N junction Provincial Road to Fofo Cahuel (42°18'39.7"S, 70°02'12.2"W).
Gastre Department: LJAMM-CNP 6046, 6048–9, Old Post area, 3 km S Provincial Road 4, 40 km W Gan Gan (42°27'43"S, 68°46'56.1"W); LJAMM-CNP 6062, 6092–4, 6103, Cerro Navidad area, 3 km S Provincial Road 4, 40 km W Gan Gan (42°24'50.8"S, 68°49'17.5"W); LJAMM-CNP 6068–9, Aguada Oveja Muerta area, 3 km S Provincial Road 4, 40 km W Gan Gan (42°26'24.6"S, 68°48'20"W); LJAMM-CNP 6084, gate at Campo Nueva Era de Santana area, 3 km S Provincial Road 4, 40 km W Gastre (42°28'25.9"S, 68°46'52"W); LJAMM-CNP 6884, Provincial Road 4, 63 km W Gan Gan (42°23'44.8"S, 68°57'54"W); LJAMM-CNP 7111, Provincial Road 4, 28.7 km E Gastre (42°23'34.5"S, 68°57'00.5"W); LJAMM-CNP 8822, Provincial Road 58, 6.7 km E junction Provincial Road 50, to Laguna Fría (42°56'15"S, 68°31'18.4"W).
Telsen Department: LJAMM-CNP 11129–30, Cañada La Leona, 19.5 km N Gan Gan (42°31"S, 68°17"W); LJAMM-CNP 11526–32, 3410, Provincial Road 67, 17.7 km N Gan Gan, 2 km detour Cañada Leona (42°24'18.8"S, 68°15'27.1"W); LJAMM-CNP 5458, Provincial Road 4, 0.8 km E Gan Gan (42°31'35"S, 68°15'29"W); LJAMM-CNP 5464, Provincial Road 4, 20.4 km E Gan Gan (42°32'08.8"S, 68°01'49.1"W); LJAMM-CNP 5506–7, 5590, Provincial Road 4, 2 km E Gan Gan (42°31'44.3"S, 68°01'06.6"W); LJAMM-CNP 5640, Provincial Road 4, 70.7 km W Telsen (42°22'55.3"S, 67°42'44.8"W); LJAMM-CNP 5667, Provincial Road 4, 65.5 km W Telsen (42°22'03.8"S, 67°39'22"W); LJAMM-CNP 6207–8, Provincial Road 67, 16.5 km N Gan Gan (42°25'06"S, 68°16'39.3"W); LJAMM-CNP 6212, Provincial Road 67, 82 km N Gan Gan (42°04'28.8"S, 68°09'13.4"W); LJAMM-CNP 6491, Provincial Road 67, 20.7 km N Gan Gan (42°24'55.77"S, 68°12'32.48"W); LJAMM-CNP 6741, Provincial Road 4, 89.5 km W Telsen, El Encuentro Ranch (42°27'27.6"S, 67°50'50"W); LJAMM-CNP 6887–93, 6960, Provincial Road 4, between 20 and 50 km E from Gan Gan (42°30'24.8"S, 67°57'33.1"W); LJAMM-CNP 7515, Provincial Road 67, to Talagapa, 53.1 km N Gan Gan (42°13'50.8"S, 68°14'23.8"W).
Literature and museum citations:
Cei (1986): cited without department. Abdala (2007): CSA 415, cited for 60 km W de Telsen. Etheridge & Christie (2003): cited only for the higher lands limiting Río Negro province. Scolaro (2005): cited for northern Chubut province.
Conservation status and phytogeographic provinces: not endangered, Provincia Patagónica and Provincia del Monte.

Liolaemus senguer ABDALA, 2005
Reference material (Fig. 12):
Paso de Indios Department: LJAMM-CNP 9038–41, Provincial Road 23, 65.1 km E junction Provincial Road 20, to Los Flamencos Ranch (44°35'50.7"S, 69°41'26.1"W).

Río Senguer Department: LJAMM-CNP 2186–8, Provincial Road 20, 23 km W Los Manantiales (45°27'22"S, 69°45'56"W); LJAMM-CNP 4577, Provincial Road 20, 5 km N Los Tamariscos (44°58'46.8"S, 70°02'53.3"W); LJAMM-CNP 4699–4701, National Road 40, 3.5 S Mayo River (45°43'27.2"S, 70°16'16.8"W); LJAMM-CNP 13067–8, National Road 40, junction National Road 260, to Lago Blanco (45°56'45.9"S, 70°24'12.5"W); LJAMM-CNP 13074–5, National Road 40, 2 km S Mayo River (45°42'57.4"S, 70°15'53"W); LJAMM-CNP 13077, National Road 40, 26.5 km N junction National Road 26, 11.5 km N entering Facundo (45°14'59.9"S, 69°56'13"W); LJAMM-CNP 13080–1, National Road 40, 26 km N La Laurita Ranch (45°22'35.01"S, 70°27'29.84"W); LJAMM-CNP 9180, National Road 26, 22.3 km NW junction Provincial Road 18 (45°57'25.5"S, 69°52'08.9"W).
Tehuelches Department: LJAMM-CNP 8895, Provincial Road 23, to Los Flamencos Ranch, 3.6 km E junction Provincial Road 20 (44°32'18.7"S, 70°21'48.2"W); LJAMM-CNP 9000–6, Provincial Road 23, 44.2 km E Provincial Road 20, to Los Flamencos Ranch, 1 km E Los Guindos Ranch (44°35'42.6"S, 69°54'06.0"W); LJAMM-CNP 9021–28, Provincial Road 23, to Los Flamencos Ranch, 3.6 km E junction Provincial Road 20 (44°32'18.7"S, 70°21'48.2"W).
Literature and museum citations:
Río Senguer Department: Abdala (2005): FML 15548–50, 26 km N of Alto Río Senguer, on National Road 40; IBA 657 (5 ej.) Los Tamariscos; FML 15739, 72 km N Mayo River; MCN 1339–40, Los Tamariscos, CSA 210–1, 26 km N Alto Río Senguer; CSA 416–8 Los Tamariscos.
Tehuelches Department: Abdala (2005): IBA 628 (6 ej.) Nueva Lubecka; IBA 526 (2 ej.) 20 km N Nueva Lubecka. Abdala (2007): FML 1603, Nueva Lubecka.
Abdala (2005): MLP S 1461–2, for Chubut Territories.
Conservation status and phytogeographic provinces: not endangered, Provincia Patagónica.

Liolaemus shehuen ABDALA, DÍAZ-GÓMEZ & JUAREZ-HEREDIA, 2012
Reference material (Fig. 10):
Telsen Department: LJAMM-CNP 6883, Provincial Road 4, 80 km W Telsen (42°24'55"S, 67°46'04"W); LJAMM-CNP 6943–52, 6961, Laguna de Vaca detour, 3.5 km S junction Provincial Road 4 (42°26'52.6"S, 67°18'51.6"W); LJAMM-CNP 11023–43, 2 km S junction Provincial Road 4, to Laguna de Vaca (42°23'20.2"S, 67°32'41.3"W).
Literature and museum citations:
Abdala et al. (2012b): Telsen Department: FML 22191–5, 22204–5, 60 km west of Telsen; FML 22217–9, 80 km west of Telsen.
Conservation status and phytogeographic provinces: not endangered, Provincia Patagónica and Provincia del Monte.

Liolaemus somuncurae CEI & SCOLARO, 1981
Reference material (Fig. 12):
Telsen Department: LJAMM-CNP 10981–8, Provincial Road 67, 11.2 km Río Negro and Chubut provinces boundary (42°04'48.9"S, 68°11'20.9"W).
Conservation status and phytogeographic provinces: data deficient, Provincia Patagónica.

Liolaemus telsen CEI & SCOLARO, 1999
Reference material (Fig. 12):
Telsen Department: LJAMM-CNP 3856–60, 3873–4, 10 km N Gan Gan, entering the first detour from Provincial Road 67 (42°26'44.9"S, 68°18'42.6"W); LJAMM-CNP 6840, Provincial Road 4, 40 km E Gan Gan (42°28'25.5"S, 67°51'54.7"W); LJAMM-CNP 6924–6, Provincial Road 67, 10 km N Gan Gan (42°26'54.3"S, 68°19'47.3"W); LJAMM-CNP 6938, Provincial Road 4, 92 km W Telsen approximately 40 km E Gan Gan (42°28'26.1"S, 67°51'35.4"W).
Literature and museum citations:

Cei & Scolaro (1999): MRSN-R 1648–1, 2, 3, 4, 5, for 80 km W Telsen city (42°22'19"S, 67°41'49"W).
Telsen Department: Abdala (2007): CSA 403–04, 80 km W Telsen; JAS 210–225 Telsen Plateau, 80 km W Telsen.
Conservation status and phytogeographic provinces: not endangered, Provincia Patagónica and Provincia del Monte.

Liolaemus uptoni SCOLARO & CEI, 2006
Reference material (Fig. 12):
Gastre Department: LJAMM-CNP 8426, Provincial Road 4, 58.3 km W Gan Gan (42°23'30.5"S, 68°56'00.1"W); LJAMM-CNP 10943–53, Bajada del Buey, Provincial Road 4, 59.3 km W Gan Gan (42°23'33.3"S, 68°56'43.4"W; Avila et al. 2007a).
Literature and museum citations:
Scolaro & Cei (2006): MACN–38742, west of Pampa Sacanana, Provincial Road 4, km 360 (42°23'46" S; 68°57'56"W).
Conservation status and phytogeographic provinces: data deficient, Provincia Patagónica.

Liolaemus xanthoviridis CEI & SCOLARO, 1980
Reference material (Fig. 12):
Escalante Department: LJAMM-CNP 10296–303, Provincial Road 27, 60 km N junction Provincial Road 25 (44°43'43.4"S, 67°54'24.0"W); LJAMM-CNP 11078–80, Provincial Road 27, 33.1 km N junction Provincial Road 25, 55.1 km NW Nollman bridge over Chico River (44°56'03.6"S, 68°01'35.8"W; Minoli & Avila 2011a).
Florentino Ameghino Department: MLP.S 2463, LJAMM-CNP 2418, FML 13051, MLP.S 2458, LJAMM-CNP 2689, Provincial Road 1, 12 km S Dos Naciones Ranch (43°46'53.48"S, 65°26'49.30"W); LJAMM-CNP 2552, Cabo Raso (44°19'23"S, 65°15'46"W); LJAMM-CNP 14341–2, Provincial Road 1, 18 km S Dos Pozos post office (44°02'07.4"S, 65°27'43.5"W).
Mártires Department: LJAMM-CNP 10304–6, Provincial Road 29, 15.5 km E junction Provincial Road 27, road to Garayalde (44°34'13.4"S, 67°39'58.4"W); LJAMM-CNP 11068–72, Provincial Road 48 (from Las Plumas to Garayalde), 86.7 km S Las Plumas, first slope to Río Chico Valley, nearby La Madreselva Ranch (44°23'54.3"S, 67°01'04.8"W); BYU 47289, BYU 47290, LJAMM-CNP 2907–9, Provincial Road 29, 45 km W Garayalde (44°33'06.9"S, 67°04'45.1"W).
Paso de Indios Department: LJAMM-CNP 10267–8, Provincial Road 27, 67.9 km N junction Provincial Road 25, between Meseta Cuadrada and Sierra Cuadrada, 7.9 km S junction Provincial Road 29 (44°40'38.7"S, 67°51'44.2"W); BYU 47282, LJAMM-CNP 2911, LJAMM-CNP 2912, BYU 46769, Bosque Petrificado, junction of Provincial Road 29 and 27, 106 km W Garayalde (44°36'15.1"S, 67°48'37.4"W).
Rawson Department: LJAMM-CNP 2201–3, MLP.S 2461, LJAMM-CNP 2485, BYU 48119, LJAMM-CNP 2487–8, 2551, Isla Escondida Bay (43°40'55"S, 65°20'23"W); LJAMM-CNP 13543–4, Old Provincial Road 1, 38.7 km S junction Provincial Road 25 (43°35'43.8"S, 65°22'59.9"W); LJAMM-CNP 14350–5, Isla Escondida beach (43°41'04.4"S, 65°20'29.2"W).
Literature and museum citations:
Gaiman Department: Abdala (2007): FML 2875, 2772, 2882, Laguna de los Indios Ranch, Road 1; FML 7937, Paleontological Park, Provincial Road 9.
Florentino Ameghino Department: Abdala (2007): for km 1533; REE 890, 4.5 km S Dos Pozos, Provincial Road 1; FML 17033, 4.5 km S Dos Pozos Provincial Road 1.
Cei & Scolaro (1980): MF 22810–12, 18 km NW Dos Pozos; FM 22805–8, Estancia Santa Clara, 25 km NW Dos Pozos; IBA-UNC 1080.1–1080.4, IBA-UNC 1081–2, 7 km from Trelew, S Río Chubut; MF 22813–4, 5–7 km S Río Chubut; MF 22815–9, 30 km NW Dos Pozos. Cei & Scolaro (1980): a

limited area in the sea shore between north of Chubut River and south of Santa Cruz bay, towards inland up to the Great Basin Laguna Salada, through the flat relief of Sotomayor Plateau. Cei (1986): from south of Chubut River to Punta Clara, limited on the W by Sotomayor Plateau.
Rawson Department: Abdala (2007): FML 8428, Estación de los Indios, Provincial Road 1; CSA 421–29, 71 km S from Rawson. Specimens examined: FBC 0001–7, Estación Paso de Indios (43°24.431'S, 65°18.099'W).
Conservation status and phytogeographic provinces: not endangered, Provincia Patagónica and Provincia del Monte.

Phymaturus calcogaster SCOLARO & CEI, 2003
Reference material (Fig. 13):
Telsen Department: LJAMM-CNP 6550–3, Provincial Road 8, Quelé Curá - Sierra Colorada (42°13'38.8"S, 66°21'41.6"W); LJAMM-CNP 6855–7, 8125–32, detour to Laguna De Vaca, 16.2 km Provincial Road 4, on the lagoon shore, passing by Elio Calfuquir Post (42°30'00"S, 67°21'35.6"W); LJAMM-CNP 13235–8, Road between Cona Niyeu and Telsen, 40.4 km junction Provincial Road 8 (42°12'10.2"S, 66°51'22.6"W).
Literature and museum citations:
Telsen Department: Scolaro et al. (2005): MACN–38609, MLP-R 5130–5, for Laguna La Vaca (42°28'45"S, 67°21'54"W).
Conservation status and phytogeographic provinces: vulnerable, Provincia Patagónica and Provincia del Monte.

Phymaturus camilae SCOLARO, JARA & PINCHEIRA-DONOSO, 2013
Reference material (Fig. 13):
Literature and museum citations:
Gastre Department: Scolaro et al. (2013): MLP-R 5586 (Holotype), MLP-R 5587–89 (Paratypes), UNCo-PH 1614, 1308 (Paratypes); JAS-DC 1316, 1318, 1320, 1609 (Paratypes), Sacanana stream bridge, adjacent to Provincial Road 4 (42°27'55.4"S, 68°43'33.3"W). Other specimens without geographic reference or locality details: JAS-DC 1306, 1307, 1309, 1317, 1321, 1581, 1587, 1957–62, 2076–2080, 2101–2118, 2125.
Conservation status and phytogeographic provinces: data deficient (due to it's recent publication and because it was not included in Abdala et al., 2012a, we consider assign the data deficient status), Provincia Patagónica.

Phymaturus castillensis SCOLARO & PINCHEIRA-DONOSO, 2010
Reference material (Fig. 13):
Literature and museum citations:
Sarmiento Department: Scolaro & Pincheira-Donoso (2010): MLP-R. 5441, Sierra del Castillo in La Juanita Ranch, adjacent to Provincial Road 24, 58 km N Sarmiento (45°08'30"S, 69°10'31"W).
Conservation status and phytogeographic provinces: vulnerable, Provincia Patagónica.

Phymaturus felixi LOBO, ABDALA & VALDECANTOS, 2010
Reference material (Fig. 13):
Literature and museum citations:
Paso de Indios Department: Lobo et al. (2010): MCN 1279–83, 108 km S of Paso de Indios, on Provincial Road 24.
Conservation status and phytogeographic provinces: vulnerable, Provincia Patagónica.

Phymaturus indistinctus CEI & CASTRO, 1973

Reference material (Fig. 13):
Río Senguer Department: MCN 810, LJAMM-CNP 2124, 2138, Provincial Road 20, Sierra de San Bernardo, 19 km W Los Manantiales (45°27'41"S, 69°42'52"W); LJAMM-CNP 2272–3, Provincial Road 20, 4 km N junction Provincial Road 22 (45°25'54"S, 69°50'25"W); MCN 1482, LJAMM-CNP 2650–1, Provincial Road 20, 4 km N junction Provincial Road 22 (45°25'54"S, 69°50'25"W); LJAMM-CNP 8198, Pampa Lehman, on Provincial Road 20 (45°24'35"S, 69°52'08"W).
Literature and museum citations:
Sarmiento Department: Lobo & Quinteros (2005a): MCN 1274–77, Las Pulgas (hills in front of Gruta de la Virgen). Cei & Castro (1973): IBA 666–1, IBA–2, IBA–3, 2 km W de Las Pulgas (Lago Musters). Scolaro (2005): nearby Lago Musters.
Río Senguer Department: Specimens examined: BYU 46736–7, Provincial Road 20, 23 km W Los Manantiales (45°27'44.6"S, 69°43'26.8"W); MCN 1481–2, Las Pulgas (45°27'61"S, 69°42'52"W).
Conservation status and phytogeographic provinces: vulnerable, Provincia Patagónica.

Phymaturus patagonicus KOSLOWSKY, 1898
Reference material (Fig. 13):
Paso de Indios Department: LJAMM-CNP 3459–64, Provincial Roads 27 and 53, in front of Cerro El Sombrero (43°50'50.1"S, 67°45'14.0"W).
Telsen Department: LJAMM-CNP 5592 Provincial Road 4, 2 km E Gan Gan (42°31'44.3"S, 68°01'06.6"W).
Literature and museum citations:
Gaiman Department: Lobo & Quinteros (2005b): SDSU 1980, 40 km WSW Dolavon; IADIZA-CH 00080, 40 km W Dolavon; IBA 789, 40 km W Dolavon; MCN 1284–1286, 40 km W Dolavon. Specimens examined: MCN 1284–6, 40 km W from Dolavon.
Paso de Indios Department: Lobo & Quinteros (2005b): IBA 783, 20 km W El Sombrero; FML 10077–85, 1 km W junction Provincial Road 53 and 90, 2.2 km SW El Sombrero Plateau; MCN 1250–58, 1261, Hills in front of El Sombrero. Specimens examined: FBC 112–6, 59 km SE Paso de Indios, Provincial Road 53 (44°07.513'S, 68°32.212'W).
Scolaro & Ibargüengoytía (2007): 40 km W Dolavon: IADIZA-CH 00080, JAS-DC 813–820, IBA-R 0789, JMC-DC 335–336, 760, 842–845, 1300, MCN-UNS 1284–87.
Telsen Department: Specimens examined: BYU 46738–9, Quelé Curá o Sierra Colorada, Provincial Road 8 (42°13'38.8"S, 66°21'41.6"W).
Lobo & Quinteros (2005b): cited for Chubut Territories MLP 777–8. Scolaro (2005): cited a vertical strip at the central eastern Chubut, with a projection from the center of this stripe to NW Chubut.
Conservation status and phytogeographic provinces: vulnerable, Provincia Patagónica and Provincia del Monte.

Phymaturus somuncurensis CEI & CASTRO, 1973
Reference material (Fig. 13):
Telsen Department: LJAMM-CNP 3407–8, LJAMM-CNP 11505–25, Provincial Road 67, 17.7 km N Gan Gan (2 km junction Cañada Leona) (42°35'41.2"S, 67°44'32.9"W); LJAMM-CNP 10977–80, Provincial Road 67, 11.2 km S Río Negro and Chubut boundary (42°04'59.9"S, 68°11'42.3"W).
Literature and museum citations:
Cei (1986): Somuncurá Plateau. Scolaro (2005): Telsen Department.
Conservation status and phytogeographic provinces: vulnerable, Provincia Patagónica.

Phymaturus videlai SCOLARO & PINCHEIRA-DONOSO, 2010
Reference material (Fig. 13):
Literature and museum citations:

Sarmiento Department: Scolaro & Pincheira-Donoso (2010): MLP-R. 5438, nearby Buen Pasto, 85 km NW from Sarmiento (45°04'11"S, 69°25'25"W).
Conservation status and phytogeographic provinces: vulnerable, Provincia Patagónica.

Figure 1

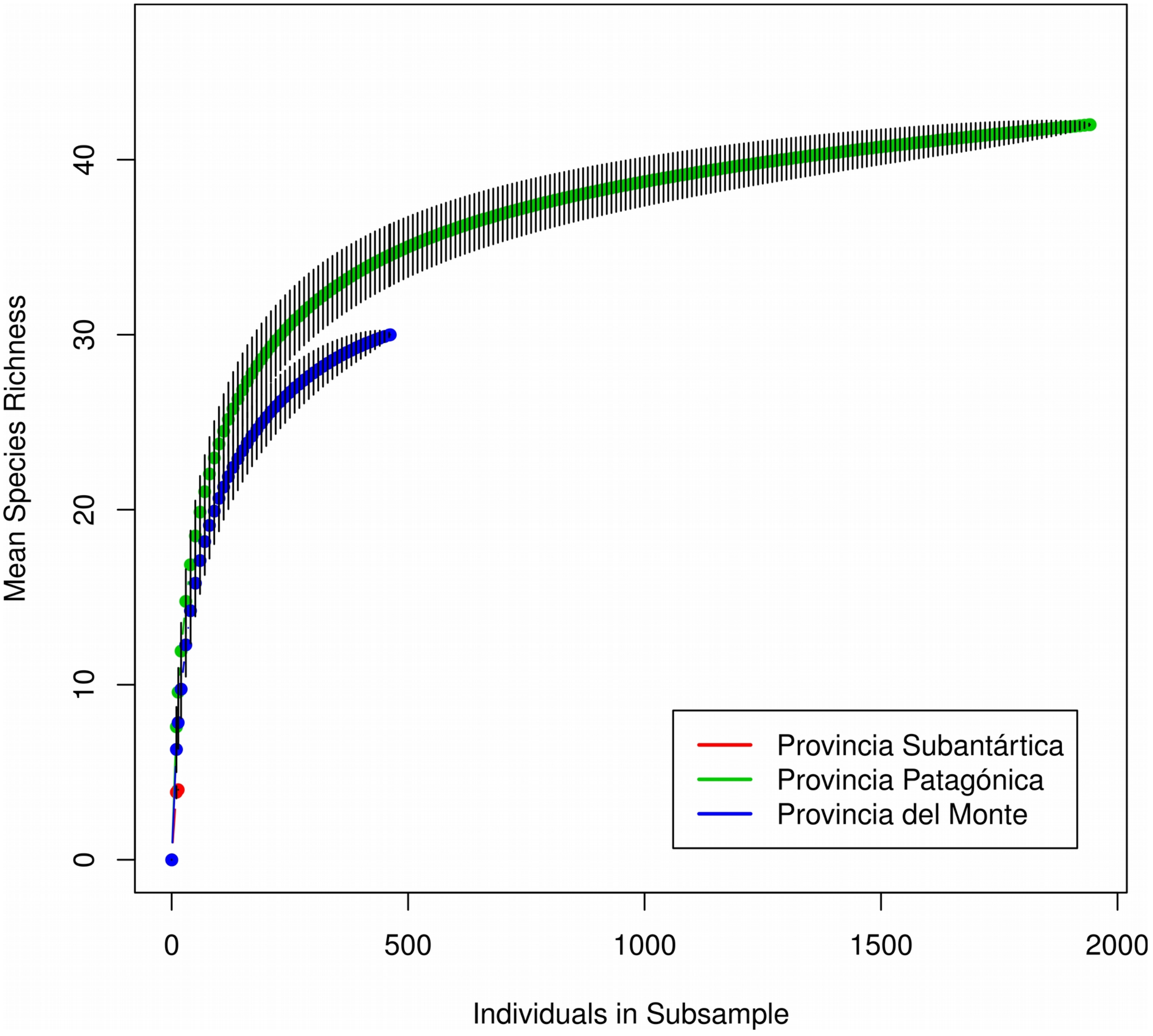

Figure 1: Rarefaction-species richness of reptiles by subsample size (number of individuals) for each phytogeographic province in Patagonia Central. Dark bars across curves show the iterative error around the mean.
